# Evaluation of Definitive Stereotactic Body Radiotherapy and Outcomes in Adults With Extracranial Oligometastasis

**DOI:** 10.1001/jamanetworkopen.2020.26312

**Published:** 2020-11-16

**Authors:** Ian Poon, Darby Erler, Roi Dagan, Kristin J. Redmond, Matthew Foote, Serena Badellino, Tithi Biswas, Alexander V. Louie, Young Lee, Eshetu G. Atenafu, Umberto Ricardi, Arjun Sahgal

**Affiliations:** 1 Sunnybrook Odette Cancer Centre, University of Toronto, Toronto, Ontario, Canada; 2 Department of Radiation Oncology, University of Florida, Jacksonville; 3 Department of Radiation Oncology and Molecular Radiation Sciences, Johns Hopkins University, Baltimore, Maryland; 4 School of Medicine, University of Queensland, Princess Alexandra Hospital, Woolloongabba, Australia; 5 Department of Oncology, University of Turin, Turin, Italy; 6 Department of Radiation Oncology, University Hospitals Cleveland Medical Center, Case Western Reserve University, Cleveland, Ohio; 7 Department of Biostatistics, University Health Network, University of Toronto, Toronto, Ontario, Canada

## Abstract

**Importance:**

The outcomes and factors that influence survival in patients with oligometastasis (OM) are not well understood and have not been well described in large-scale studies.

**Objective:**

To evaluate overall progression-free survival (PFS), widespread progression (WSP) outcomes, and survival factors from a pooled data set of 1033 patients with OM treated with stereotactic body radiotherapy (SBRT).

**Design, Setting, and Participants:**

Case series from January 1, 2008, to December 31, 2016. The dates of analysis were April 2019 to May 2020. The setting was multi-institutional tertiary care hospitals. Participants were consecutive patients with 5 or fewer extracranial OMs whose primary tumor was treated curatively.

**Exposure:**

Definitive SBRT.

**Main Outcomes and Measures:**

Overall survival (OS), progression-free survival, rate of WSP, patterns of failure, and factors altering OS.

**Results:**

In the largest international OM case series to date (1033 participants) (mean age, 68.0 years [range, 18.0-94.3 years]; 601 [58.2%] men), 1416 SBRT courses were delivered to patients with 1 OM (596 [57.7%]), 2 OMs (245 [23.7%]), 3 OMs (105 [10.2%]), 4 OMs (55 [5.3%]), and 5 OMs (32 [3.1%]). The median follow-up was 24.1 months (range, 0.3-104.7 months), and the median OS was 44.2 months (95% CI, 39.2-48.8 months). The median PFS was 12.9 months (95% CI, 11.6-14.2 months), and the median time to WSP was 42.5 months (95% CI, 36.8-53.5 months). The OS rates were 84.1% (95% CI, 81.7%-86.2%) at 1 year, 56.7% (95% CI, 53.0%-60.2%) at 3 years, and 35.2% (95% CI, 30.1%-40.3%) at 5 years. The 3-year OS, PFS, and WSP rates were 56.7% (95% CI, 53.0%-60.2%), 23.0% (95% CI, 20.2%-25.9%), and 45.2% (95% CI, 41.4%-48.9%), respectively. The 5-year OS, PFS, and WSP rates were 35.2% (95% CI, 30.1%-40.3%), 14.8% (95% CI, 11.9%-17.9%), and 54.5% (95% CI, 49.8%-59.2%), respectively. At the time of first progression, 342 patients (33.1%) had recurrence of OM disease, and 230 patients (22.3%) underwent subsequent ablative therapies to all known metastatic sites. Multivariable analyses identified primary tumor type (hazard ratio [HR], 3.73; 95% CI, 1.75-7.94; *P* < .001 for breast; 5.75; 95% CI, 2.88-11.46; *P* < .001 for colorectal; 4.67; 95% CI, 2.12-10.31; *P* < .001 for kidney; 10.61; 95% CI, 5.36-20.99; *P* < .001 for lung; and 12.00; 95% CI, 6.06-23.76; *P* < .001 for other [with prostate being the reference group]), metachronous OM presentation more than 24 months since initial diagnosis (HR, 0.63; 95% CI, 0.49-0.80; *P* < .001), metastases confined to the lung only (HR, 0.58; 95% CI, 0.48-0.72; *P* < .001), and nodal or soft-tissue metastases only (HR, 0.49; 95% CI, 0.26-0.90; *P* = .02) as survival factors. Sixty-six (6.4%) grade 3 or higher toxic effects were observed, including 1 (0.1%) grade 5 event.

**Conclusions and Relevance:**

This study found favorable long-term OS and WSP rates associated with extracranial OM ablated with SBRT; however, modest PFS rates were observed. A substantial proportion of patients with OM developed progressive disease and were treated with local ablation. Factors that can inform clinical decision-making and clinical trial design include primary tumor type, a metachronous presentation more than 24 months since diagnosis, and the site of OM presentation.

## Key Points

**Question:**

What are survival outcomes and factors specific to patients with oligometastasis (OM) treated with stereotactic body radiotherapy?

**Findings:**

In the largest international OM case series to date (1033 participants), the median overall survival was prolonged at 44.2 months and statistically significantly associated predominantly with primary tumor type and a metachronous presentation more than 24 months since initial OM diagnosis. The median progression-free survival was 12.9 months; however, a substantial proportion of patients relapsed in an oligoprogressive pattern that was amenable to repeat local therapy.

**Meaning:**

Findings of this study suggest that patients with OM disease have long-term overall survival and modest progression-free survival; a metachronous presentation more than 24 months since diagnosis is favorable.

## Teaser

This case series reports overall progression-free survival, widespread progression outcomes, and prognostic survival factors from a pooled data set of 1033 patients with oligometastasis treated with stereotactic body radiotherapy.

## Introduction

The historical results after surgical metastatectomy in select patients with solitary metastases^1,2^ and more recently using stereotactic body radiotherapy (SBRT) for patients with oligometastasis (OM) suggest the potential for cure based on a small population of long-term survivors from both of these scenarios.^3^ Skepticism remains as to the contribution of local ablative treatment, as opposed to the impact of effective systemic therapies or simply an indolent natural cancer history.^4^ Clinical trials have reported overall survival (OS) or progression-free survival (PFS) benefits specific to localized metastatic tumor ablation.^5,6,7,8^ However, the evidence is limited to small phase 2 randomized clinical trials (RCTs),^6,8,9,10^ with no confirmatory phase 3 RCTs to date and no robust large series reporting detailed outcomes. Despite the lack of evidence, patients are increasingly receiving ablative technologies to sites of OM, in part because of the availability of nonsurgical modalities, such as SBRT.^11^

Stereotactic body radiotherapy is a nonsurgical ablative treatment modality that has become part of mainstream practice. It has become a dominant treatment option for managing OM^12^ given the high rates of local control, favorable toxic effect profiles, virtually unrestricted delivery to multiple anatomic sites, and a purported synergistic association with immunotherapy.^13^ There is an urgent need not only for RCTs to prove SBRT utility but also for large-scale outcome-based studies to inform practice. Therefore, the objective of this international multi-institutional retrospective case series was to report OS, PFS, and the cumulative incidence of widespread progression (WSP) in patients whose primary tumor was radically treated with curative intent and up to 5 extracranial OMs treated with SBRT. We specifically excluded patients presenting with intracranial brain metastases.

## Methods

In this retrospective case series, an international consortium consisting of 6 high-volume academic radiation oncology centers—the Sunnybrook Odette Cancer Centre of the University of Toronto, University of Florida, Johns Hopkins University, Princess Alexandra Hospital at the University of Queensland, University of Turin, and the University Hospitals Cleveland Medical Center—pooled their collective consecutive cases of patients with extracranial OM treated with SBRT from January 1, 2008, to December 31, 2016. Institutional research board approval at all sites was obtained, with appropriate data-sharing agreements. The requirement to obtain informed consent was waived at the study coordinating site (Sunnybrook Odette Cancer Centre) because it was determined that the study involved no more than minimal risk to the participants and the lack of consent was unlikely to adversely affect their welfare. Each center was selected according to an academic presence in the field of SBRT and the ability to identify consecutive patients treated with SBRT for OM. This study followed the Strengthening the Reporting of Observational Studies in Epidemiology (STROBE) reporting guideline. The dates of analysis were April 2019 to May 2020.

To maximize consistency of data collection, a Microsoft Access (Microsoft Corp) database and a detailed associated data dictionary (eMethods in the Supplement) were created with predefined fields developed by all authors. Patient data were deidentified and transferred to the study coordinating site (Sunnybrook Odette Cancer Centre), with data quality assurance procedures performed before analysis (eFigure in the Supplement). Inclusion criteria consisted of patients 18 years or older with a pathologically confirmed cancer diagnosis, radical curative-intent treatment delivered to the primary tumor, and development of OM. The OM state was defined as 5 or fewer extracranial metastases presenting either synchronously (OM disease developing within 6 months of diagnosis) or metachronously (OM disease developing beyond 6 months of diagnosis). Patients enrolled in clinical trials were included in this cohort if no more than 1 metastatic site was left untreated, with all other sites receiving either definitive radiotherapy or SBRT. Patients with prior non-SBRT treatments were not excluded if at least 1 OM had been treated with SBRT during the study period. For example, if a patient had surgical metastatectomy before SBRT to a separate site, that metastasis was included in the total baseline metastatic count. Patients who were downstaged to an OM state were excluded, as were those with brain metastases and a primary hematologic, central nervous system, or germ cell tumor.

Complete baseline staging was required (computed tomography or magnetic resonance imaging of the brain, computed tomography of the chest or abdomen or pelvis, or a positron emission tomographic scan) within 4 months of the first SBRT treatment to confirm an OM state. Malignant lymph nodes, defined by site-specific size criteria or positron emission tomography avidity, were counted as an OM site (or sites). Each involved lymph node was counted as a separate OM. For the purposes of this study, SBRT was defined in a practical fashion as treatment given in 15 fractions or fewer using an SBRT technique, with a fractionation scheme that would not otherwise be considered palliative (ie, 4 Gy by 5 fractions, 3 Gy by 10 fractions, or 8 Gy by 1 or 2 fractions).

### Treatment and Follow-up

The SBRT protocols for each institution with respect to simulation, immobilization, dose fractionation, and image guidance have been previously described.^14^ The follow-up protocols after SBRT were consistent among the institutions (every 2-4 months in the first year, every 3-6 months in the second year, every 4-6 months in the third and fourth years, and every 6-12 months in the fifth year and thereafter). However, imaging modalities varied depending on the treatment site and institution. At a minimum, centers used complete computed tomographic chest, abdomen, pelvis, and brain imaging to restage and identify local tumor control and distant progression, with additional studies (eg, magnetic resonance imaging for liver, spine, and nonspine bone metastases) per institutional practice.^15^ Positron emission tomography was not consistently used. Toxic effects were retrospectively classified using the National Cancer Institute Common Toxicity for Adverse Events (version 4) and were categorized as acute if they occurred within 3 months of SBRT and as late if more than 3 months after SBRT. Adverse events were recorded only if they met a minimum grade of 3 according to the National Cancer Institute Common Toxicity for Adverse Events.

### Outcomes

Categorical variables of patients’ baseline and disease characteristics were summarized with counts and percentages. Continuous variables were summarized with medians and ranges. The primary objective was to identify rates of OS. Secondary objectives included PFS rates and progression defined at the date of radiologic evidence of either local progression, new metastases, death, or last follow-up for patients who remained alive. Widespread progression is a novel metric that we defined as the time to further metastatic dissemination, such that the patient would not be amenable to any further local ablative therapy inclusive of developing at least 6 new sites of extracranial metastases or malignant effusions.

### Statistical Analysis

Overall survival rates and progression statistics were calculated with the Kaplan-Meier method using the start date of SBRT to the first OM site treated, and comparisons were assessed with the log-rank test. Cumulative incidence of WSP rates was calculated using a competing risk analysis. Univariable and multivariable Cox proportional hazards regression modeling was performed to identify survival factors. Variables with *P* ≤ .20 on univariable analysis were entered into the multivariable regression model and sequentially removed using backward stepwise elimination until the remaining covariates had at least *P* < .05. Variables identified as survival factors from the final multivariable analysis were then used as stratification factors for Kaplan-Meier survival curves for illustrative purposes. All tests were 2-tailed, and results were considered statistically significant at *P* < .05. Statistical analyses were performed using SAS, version 9.4 (SAS Institute Inc).

## Results

Participants were consecutive patients with 5 or fewer extracranial OMs whose primary tumor was treated curatively. Between January 1, 2008, and December 31, 2016, a total of 1033 treated patients (mean age, 68.0 years [range, 18.0-94.3 years]; 601 [58.2%] male and 432 [41.8%] female) met the inclusion criteria.

Overall, 596 of 1033 patients (57.7%) had 1 OM, 245 patients (23.7%) had 2 OMs, 105 patients (10.2%) had 3 OMs, 55 patients (5.3%) had 4 OMs, and 32 patients (3.1%) had 5 OMs. Sunnybrook Odette Cancer Centre contributed 398 patients (38.5%), University of Florida contributed 246 patients (23.8%), University of Turin contributed 117 patients (11.3%), Johns Hopkins University contributed 96 patients (9.3%), Princess Alexandra Hospital contributed 94 patients (9.1%), and University Hospitals Cleveland Medical Center contributed 82 patients (7.9%). Baseline patient, tumor, and treatment details are summarized in Table 1. The median follow-up was 24.1 months (range, 0.3-104.7 months).

**Table 1.** Patient Demographic Characteristics

Variable	Patients, No. (%) (N = 1033)
Age, median (range), y	68.0 (18.0-94.3)
Sex	
Male	601 (58.2)
Female	432 (41.8)
Primary tumor type	
Breast	84 (8.1)
Colorectal	235 (22.7)
Kidney	63 (6.1)
Lung	260 (25.2)
Prostate	132 (12.8)
Melanoma	37 (3.6)
Sarcoma	36 (3.5)
Head and neck	47 (4.5)
Thyroid	11 (1.1)
Pancreas	28 (2.7)
Hepatic or biliary	18 (1.7)
Gynecologic	19 (1.8)
Other gastrointestinal	18 (1.7)
Other genitourinary	17 (1.6)
Unknown	5 (0.5)
Other	23 (2.2)
Histology	
Adenocarcinoma	589 (57.0)
Squamous cell	129 (12.5)
Ductal carcinoma	67 (6.5)
Renal cell	63 (6.1)
Sarcoma	36 (3.5)
Melanoma	37 (3.6)
Other	86 (8.3)
Unknown	26 (2.5)
Metastatic presentation	
Synchronous	279 (27.0)
Metachronous	754 (73.0)
Metastatic interval (range), mo	17.3 (0-293.0)
No. of metastases	
1	596 (57.7)
2	245 (23.7)
3	105 (10.2)
4	55 (5.3)
5	32 (3.1)
No. of organs involved	
1	875 (84.7)
2	140 (13.6)
3-4	18 (1.7)
Prior definitive metastasis-directed therapy	
No	805 (77.9)
Yes	228 (22.1)
Prior systemic therapy for metastatic disease	
No	665 (64.4)
Yes	368 (35.6)
Type of prior systemic therapy for metastatic disease	
Chemotherapy	218 (21.1)
Hormone therapy	134 (13.0)
Target therapy	65 (6.3)
Immunotherapy	14 (1.4)
All known sites of disease treated	
No	49 (4.7)
Yes	981 (95.0)
Unknown	3 (0.3)
Prior systemic therapy for primary disease	
No	477 (46.2)
Yes	555 (53.7)
Unknown	1 (0.1)
Type of prior systemic therapy for primary disease	
Chemotherapy	431 (41.7)
Hormone therapy	134 (13.0)
Target therapy	65 (6.3)
Immunotherapy	14 (1.4)
Not specified	5 (0.5)

**Figure 1.**
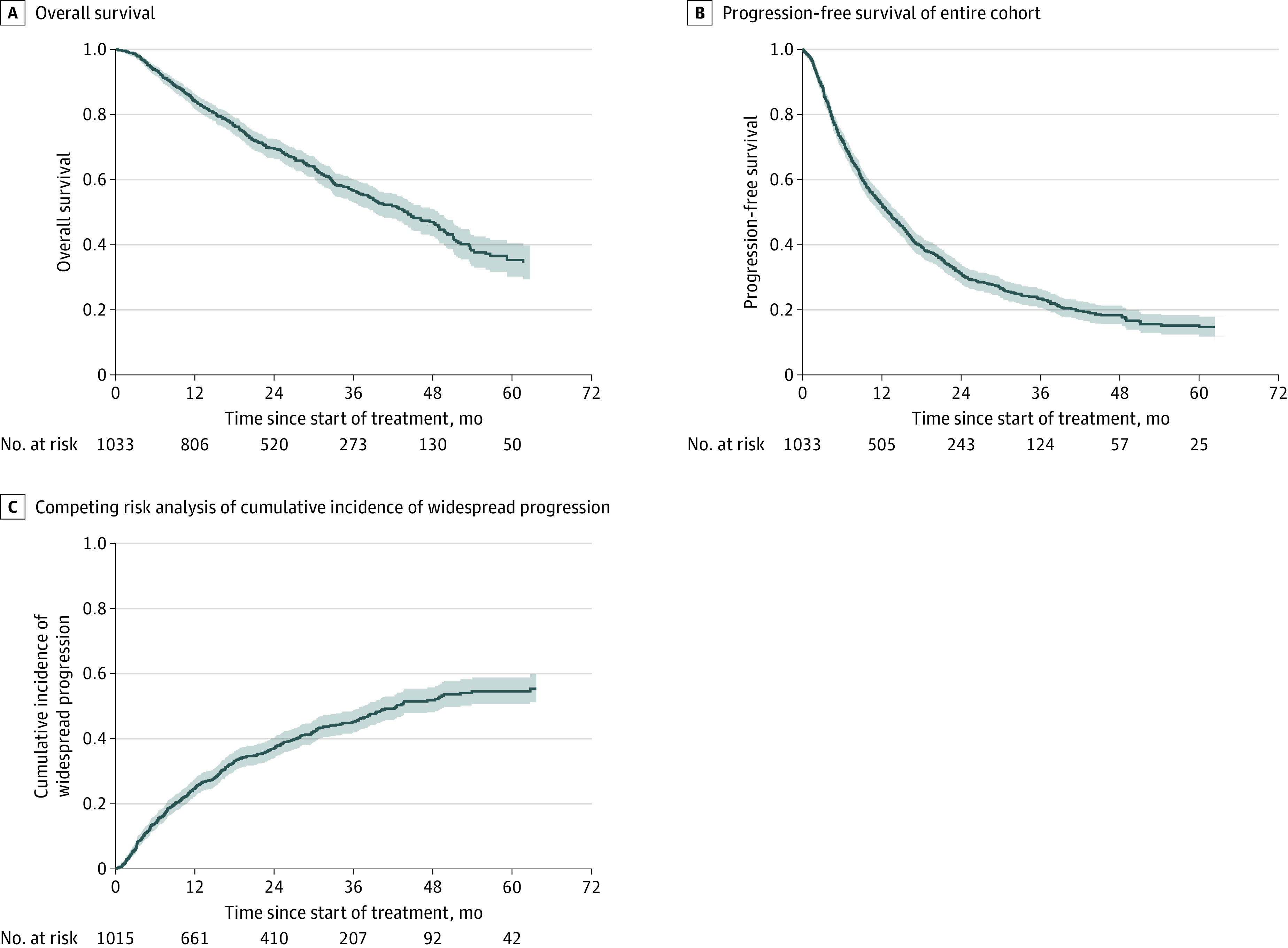
Kaplan-Meier Estimates A-C, Data are shown for overall survival and progression-free survival in the entire cohort and for widespread progression among 1015 patients. Shading indicates 95% CIs.

Main outcomes and measures were OS, PFS, rate of WSP, patterns of failure, and factors altering OS. In total, 1416 SBRT courses were delivered at presentation to OM sites (eTable 1 in the Supplement). Among 1033 patients, most metastases (929 [89.9%]) involved a single organ (lung in 414 [44.6%], bone in 277 [29.8%], and liver in 124 [13.3%]), whereas 2-organ and 3-organ combinations represented only 95 cases (9.2%) and 9 cases (0.9%), respectively (eTable 2 in the Supplement). By the end of the study period, 433 patients (41.9%) had died, resulting in a median OS for the entire cohort of 44.2 months (95% CI, 39.2-48.8 months). The OS rates were 84.1% (95% CI, 81.7%-86.2%) at 1 year, 69.6% (95% CI, 66.5%-72.5%) at 2 years, 56.7% (95% CI, 53.0%-60.2%) at 3 years, and 35.2% (95% CI, 30.1%-40.3%) at 5 years (Figure 1A).

Covariates considered for univariable analysis and multivariable analysis are summarized in Table 2. Multivariable analyses identified the following statistically significant survival factors: primary tumor type, metachronous OM presentation more than 24 months since initial diagnosis vs synchronous or metachronous presentation 24 months or less since diagnosis (hazard ratio [HR], 0.63; 95% CI, 0.49-0.80; *P* < .001), OMs confined to the lung only (HR, 0.58; 95% CI, 0.48-0.72; *P* < .001), and OMs confined to nodal or soft-tissue metastases only (HR, 0.49; 95% CI, 0.26-0.90; *P* = .02).

**Table 2.** Univariable and Multivariable Analyses of Overall Survival

Variable	Univariable		Multivariable	
	HR (95% CI)	*P* value	HR (95% CI)	*P* value
Age	1.00 (1.00-1.01)	.29	NA	NA
Sex	1.01 (0.84-1.23)	.88	NA	NA
Primary tumor type				
Prostate	1 [Reference]	NA	1 [Reference]	NA
Breast	3.95 (1.86-8.40)	<.001	3.73 (1.75-7.94)	<.001
Colorectal	5.33 (2.69-10.57)		5.75 (2.88-11.46)	
Kidney	4.49 (2.04-9.88)		4.67 (2.12-10.31)	
Lung	9.13 (4.66-17.90)		10.61 (5.36-20.99)	
Other	10.43 (5.31-20.48)		12.00 (6.06-23.76)	
Metastatic presentation				
Synchronous	1 [Reference]	NA	1 [Reference]	NA
Metachronous ≤24 mo	1.06 (0.85-1.33)	<.001	0.99 (0.78-1.26)	.95
Metachronous >24 mo	0.53 (0.41-0.67)	<.001	0.63 (0.49-0.80)	<.001
Single or multiple metastases	1.03 (0.85-1.24)	.78	NA	NA
Single or multiple organ	1.11 (0.86-1.43)	.44	NA	NA
Lung metastases only	0.87 (0.72-1.06)	.16	0.58 (0.48-0.72)	<.001
Bone metastases only	0.75 (0.60-0.95)	.02	NA	NA
Liver metastases only	1.75 (1.35-2.27)	<.001	NA	NA
Adrenal metastases only	2.45 (1.58-3.81)	<.001	NA	NA
Nodal or soft-tissue metastases only	0.57 (0.31-1.03)	.06	0.49 (0.26-0.90)	.02
Potential second primary	1.21 (0.95-1.55)	.13	NA	NA
Prior definitive metastasis-directed therapy	1.02 (0.82-1.27)	.98	NA	NA
Prior systemic therapy for metastatic disease	0.86 (0.70-1.05)	.15	NA	NA
Time from metastases to SBRT	1.00 (0.99-1.00)	.09	NA	NA

Abbreviations: HR, hazard ratio; NA, not applicable; SBRT, stereotactic body radiotherapy.

**Figure 2.**
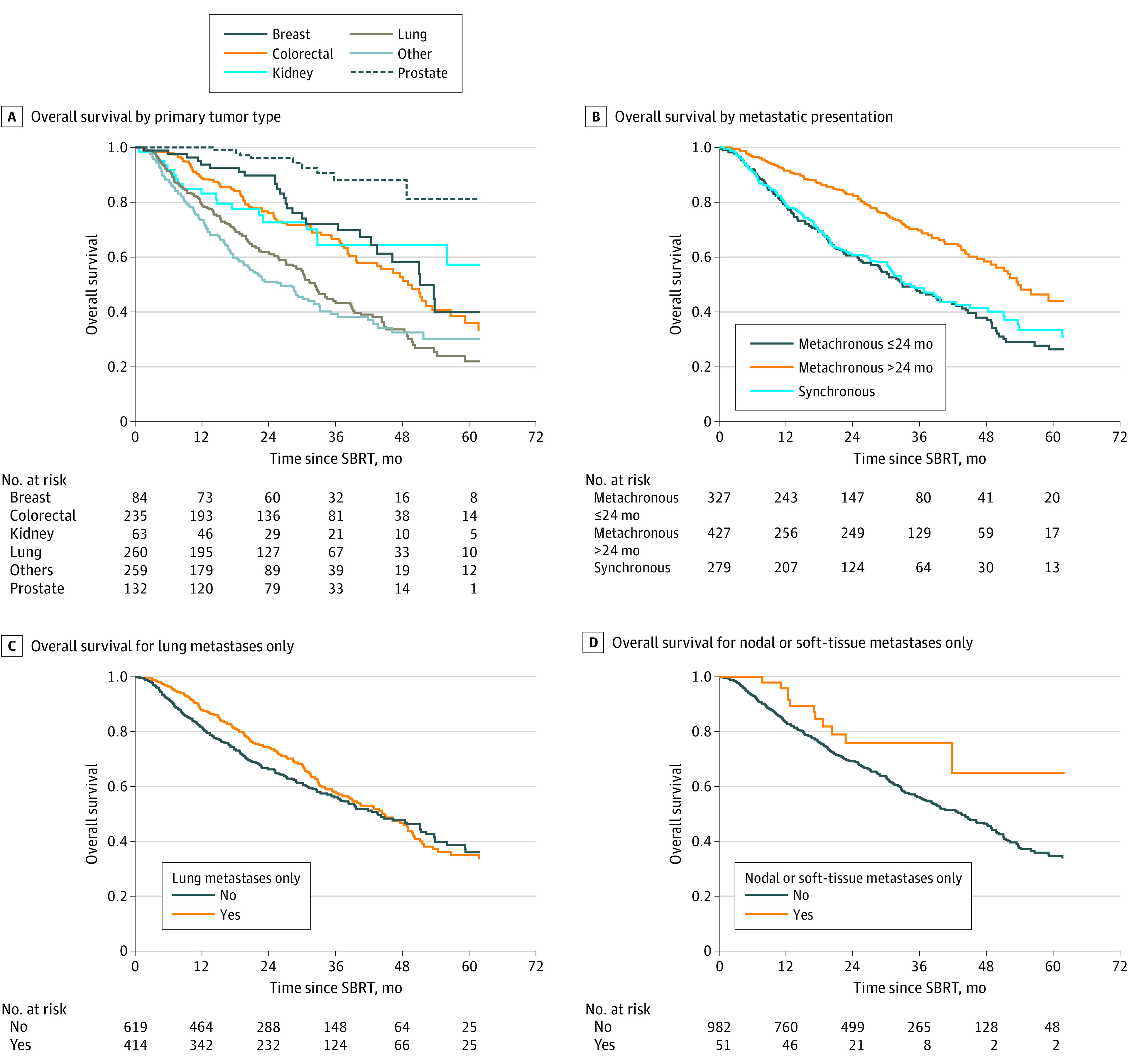
Overall Survival A-D, Data are shown for the entire cohort. SBRT indicates stereotactic body radiotherapy.

The median OS for 132 patients with prostate cancer was not reached during the study period, with 1-year, 2-year, 3-year, and 5-year OS rates of 100% (95% CI, 100%-100%), 96.0% (95% CI, 89.8%-98.5%), 87.9% (95% CI, 76.1%-94.1%), and 81.2% (95% CI, 60.4%-91.7%), respectively, whereas the median OS for the other cohorts was 82 months (95% CI, 32.7-infinity months) for kidney cancer, 51 months (95% CI, 42.4-infinity months) for breast cancer, 49 months (95% CI, 39.9-56.7 months) for colorectal cancer, 32 months (95% CI, 28.8-38.5 months) for lung cancer, and 26 months (95% CI, 20.3-32.2 months) for other cancers (Figure 2A). For kidney and breast cancers, upper CI levels could not be calculated because there was an insufficent number of events to estimate the SE. The median OS for 279 patients with synchronous disease and for 327 patients with metachronous disease presenting within 24 months of their initial diagnosis was 33.2 months (with HR used as the reference) and 33.0 months (HR, 0.99; 95% CI, 0.78-1.26; *P* = .95), respectively, compared with 53.6 months (HR, 0.63; 95% CI, 0.49-0.80; *P* < .001) for 427 patients with metachronous disease presenting more than 24 months since initial diagnosis (*P* < .001) (Figure 2B). More specifically, the 1-year, 2-year, 3-year, and 5-year OS rates for the patients with metachronous OM developing more than 24 months since initial diagnosis were 91.5%, 82.7%, 69.6%, and 43.8%, respectively. These were significantly prolonged compared with the 1-year, 2-year, 3-year, and 5-year OS rates in the synchronous cohort at 79.1%, 60.7%, 48.4% and 33.4%, respectively, and 78.9%, 60.4%, 47.5%, and 26.2% in the patients presenting within 24 months of their primary tumor diagnosis, respectively. The median OS was 44.8 months (95% CI, 38.5-49.7 months) for 414 patients presenting solely with lung metastases vs 43.5 months (95% CI, 37.5-51.2 months) for 619 patients with OM involving other organ sites (Figure 2C). The median OS for 51 patients with nodal or soft-tissue metastases only was not reached vs 43.5 months (*P* = .05) for 982 patients with OM involving other organ sites (Figure 2D). Patients in whom a lung biopsy confirmed an OM state did not appear to be statistically significantly different from those without histological confirmation of an OM state in the univariable analysis. Further statistical details are listed in eTable 3 in the Supplement.

At the time of analysis, 766 of 1033 patients (74.2%) had progression of their disease (Figure 1B). The median PFS was 12.9 months (95% CI, 11.6-14.2 months), and PFS rates were 52.1% (95% CI, 48.9%-55.1%) at 1 year, 30.8% (95% CI, 27.8%-33.8%) at 2 years, 23.0% (95% CI, 20.2%-25.9%) at 3 years, and 14.8% (95% CI, 11.9%-17.9%) at 5 years. To further describe the patterns of progression, the median time to WSP was 42.5 months (95% CI, 36.8-53.5 months), with WSP rates of 24.6% (95% CI, 21.8%-27.4%) at 1 year, 37.0% (95% CI, 33.7%-40.4%) at 2 years, 45.2% (95% CI, 41.4%-48.9%) at 3 years, and 54.5% (95% CI, 49.8%-59.2%) at 5 years (Figure 1C). Among 446 of 1033 patients (43.2%) developing WSP, the median time to WSP was 10.8 months (range, 0.20-72.3 months).

At the time of first progression, 342 patients (33.1%) had recurrence of OM disease, 220 patients (21.3%) had developed WSP, and 143 patients (13.8%) experienced failure within a previously treated OM site. For the 220 of 705 patients (31.2%) with WSP, the median time to progression was 5.7 months. Of 328 patients (31.8%) without recurrence during the study period, 267 patients (25.8%) were alive and disease free with a median follow-up of 26.3 months (range, 1.6-89.2 months), whereas 67 died of intercurrent illness, with no evidence of disease progression. Of 342 patients who had recurrence of OM disease at first progression, 230 patients (22.3% of the entire case series) underwent subsequent ablative therapies to all known metastatic sites.

The crude treated metastasis control rate after SBRT to all sites of OM was 79.2% (1079 of 1416). Site-specific crude organ-specific outcomes were 88.1% (171 of 194) for nonspine bone OM, 83.6% (97 of 116) for nodal or soft-tissue OM, 79.5% (515 of 648) for lung OM, 72.9% (35 of 48) for adrenal OM, 74.2% (167 of 225) for spine OM, and 63.8% (118 of 185) for liver OM.

Sixty-six (6.4%) grade 3 or higher acute and late toxic effects were observed (eTable 4 in the Supplement). Of 3 patients with bile duct stenosis (2 cases attributed to liver SBRT and 1 case after nodal SBRT), 1 patient died; the death was attributed to SBRT (a grade 5 event [0.1%]). Respiratory-related complications from lung SBRT were most common, with pneumonitis and shortness of breath or cough representing 28 (2.7%) of the reported toxic effects.

## Discussion

We present outcomes for 1033 patients with OM treated with SBRT from 6 academic institutions, representing the largest international OM case series to date. Inclusion criteria were limited to patients having their primary tumor treated curatively and presenting with up to 5 extracranial OMs. For the entire cohort, the median OS was 44.2 months, and the median PFS was 12.9 months. To further describe the pattern of progression after SBRT, a novel parameter termed WSP was used, and a median time to WSP of 42.5 months was observed. In addition, the adverse event profile was favorable, with 1 grade 5 serious adverse event (0.1%) and 66 grade 3 or higher serious adverse events (6.4%) (eTable 4 in the Supplement). Multivariable analysis identified the following 4 statistically significant survival factors for OS: primary tumor type, metachronous OM presentation more than 24 months since initial diagnosis, metastases confined to the lung only, and nodal or soft-tissue metastases only (Table 2).

The association of primary tumor type and OS (Figure 2A) aligns with other results in the OM literature. The longest OS rates in the present series were observed in patients with prostate OM, with a 3-year OS rate of 87.9%, and the median OS was not reached. These prostate cancer–specific rates are similar to those in the Systemic Therapy for Advanced or Metastatic Prostate Cancer: Evaluation of Drug Efficacy (STAMPEDE) trial,^16^ which reported a 3-year OS rate of 81% in patients with prostate cancer with a low metastatic burden in whom radical radiotherapy was directed only to the prostate primary. In that trial, metastatic sites received the standard of care along with lifelong androgen deprivation therapy with or without docetaxel. A 3-year OS improvement of 8% was seen in the patients with low metastatic burden who received prostate radiotherapy, raising the question of whether delivering ablative doses to all metastatic deposits may further improve survival results.^17^ However, successfully testing the value of SBRT to OM sites may prove difficult because of the excellent long-term survival in OM prostate cancer that is largely owing to the availability of highly effective systemic therapies. In addition, these data highlight the need for caution when interpreting trials of mixed tumor sites of SBRT for OM without stratification of patients with prostate cancer or favorable anatomic sites of OM.^18^ Ultimately, disease-specific phase 3 RCTs are needed and ongoing.^19^

As recently highlighted by the European Society for Radiotherapy and Oncology and European Organisation for Research and Treatment of Cancer consensus document on OM disease,^20^ a gap in the OM evidence exists in defining the importance of the timing of OM disease presentation. The present study provides evidence to support a cutoff of 24 months, such that a metachronous presentation more than 24 months since initial OM diagnosis was analogous to a synchronous presentation (Table 2 and Figure 2B). Figure 2B shows the virtual overlap in the OS curves for the synchronous cohort and the cohort with metachronous disease presenting within 24 months of initial OM diagnosis, with a median OS of approximately 33 months for both cohorts. These results were statistically significantly different from the median OS of 53.6 months (HR, 0.63; 95% CI, 0.49-0.80) in the cohort with metachronous disease presenting more than 24 months since initial OM diagnosis. These data should be incorporated into stratification criteria and survival estimates for future trials.

The analysis also identified the importance of the site of OM for metastases confined to the lung only (HR, 0.58) and for nodal or soft-tissue metastases only (HR, 0.49) as statistically significant factors associated with OS (Figure 2C and D). The improved median OS difference for patients presenting solely with lung metastases was small at 1.3 months vs all other anatomic sites of OM, and we surmise that the large sample size of the cohort with lung metastases only (n = 414) likely resulted in the significance observed (*P* < .001). Although a statistically significant result, only 51 patients were seen with the favorable OM presentation involving nodal or soft-tissue metastases only, and this result remains to be validated.

Table 3 summarizes the limited number of reported phase 2 RCTs specific to the therapeutic impact of SBRT on OM. Notably, only 239 patients were randomized across the 4 trials.^6,8,9,10^ The randomized phase 2 Stereotactic Ablative Radiotherapy for the Comprehensive Treatment of Oligometastases (SABR-COMET) trial has recently been updated.^8^ That trial included various primary tumors, including patients with prostate cancer, and patients were randomized to consolidation SBRT vs standard of care. A statistically significant OS advantage was found in the group randomized to SBRT vs the standard of care group, with a median OS of 50 months vs 28 months (and a doubling of PFS from 5.4 months to 11.6 months). The overall median OS and PFS of 44.2 months and 12.9 months, respectively, in the present study provide comparative evidence supporting those outcomes in the consolidation SBRT experimental group. Notably, a phase 2 RCT by Gomez et al^6^ of 49 patients with non–small cell lung cancer and OM also reported improved OS and PFS at 41.2 months and 14.2 months, respectively, after consolidative SBRT (compared with 17.0 months and 4.4 months, respectively, in the standard of care group). Furthermore, in the subcohort of 260 patients with a lung cancer primary tumor in the present study, the median OS and PFS rates were 32.4 months and 15.7 months, respectively.

**Table 3.** Summary of Phase 2 RCTs Using SBRT in the Setting of Oligometastasis, With Reference to the Present Series

Source	Sample size	Tumor type	Study design	Results
Gomez et al^6^	49	NSCLC	Phase 2 RCT, multi-institutional; 1:1 maintenance systemic therapy or observation vs local consolidative therapy to all disease sites	OS 41.2 vs 17.0 mo (*P* = .02); PFS 14.2 vs 4.4 mo (*P* = .02)
Iyengar et al^10^	29	NSCLC, *EGFR/ALK* negative	Phase 2 RCT, single institution; 1:1 maintenance chemotherapy vs SABR to all disease sites, followed by maintenance chemotherapy	PFS 9.7 vs 3.5 mo (*P* = .01)
SABR-COMET^8^	99	Any	Phase 2 RCT, multi-institutional; 1:2 standard of care vs standard of care plus SABR to all sites of disease	OS 41 vs 28 mo (*P* = .09); PFS 12 vs 6 mo (*P* = .001)
Ost et al^9^	62	Prostate	Phase 2 RCT, multi-institutional; 1:1 ADT vs SBRT to all metastatic disease sites	ADT-free survival 21 vs 13 mo (*P* = .11)
Present series	1033	Any	Retrospective, multi-institutional; SBRT to metastatic sites, with definitive primary disease treatment	OS 44 mo; PFS 12.9 mo

Abbreviations: ADT, androgen deprivation therapy; NSCLC, non–small cell lung cancer; OS, overall survival; PFS, progression-free survival; RCT, randomized clinical trial; SABR, stereotactic ablative radiotherapy; SABR-COMET, Stereotactic Ablative Radiotherapy for the Comprehensive Treatment of Oligometastases; SBRT, stereotactic body radiotherapy.

With a median PFS of 12.9 months and 2-year and 5-year PFS rates of 30.8% and 14.8%, respectively, most of the population with OM in the present study continued to relapse within a short time frame. Data beyond this end point provide insight into the OM clinical journey. On first progression, 342 patients (33.1%) had recurrence of OM disease, with a second course of local ablative therapy to 230 patients (22.3%). These results are in line with the randomized phase 2 data by Ost et al,^9^ in which 11 of 31 hormone-naive patients with prostate cancer (35.5%) and 6 of 25 patients (24.0%) with non–small cell lung cancer received a second course of local ablative therapy.

The reporting of the incidence of WSP in the present study also provides novel insight into the patterns of progression after SBRT. For the entire cohort, an overall median time to WSP of 42.5 months was observed, and this finding suggests that WSP is not an early pattern of progression. However, of the 446 of 1033 patients (43.2%) who developed WSP, the median time to developing WSP was only 10.8 months. For those who progressed, 220 of 705 (31.2%) presented with widespread disease, with a short median WSP time of 5.7 months. This result highlights the ongoing challenge of defining the optimal patient cohort that can benefit from upfront SBRT vs systemic therapy. Advanced imaging techniques and blood-based or tissue-based biomarkers, such as circulating cell-free DNA, may improve cohort definition and inform treatment decisions.^18,19^ Among 328 patients in the present study with no reported recurrence during the study period, 267 (25.8% of 1033) were alive and disease free at a median follow-up of 26.3 months (range, 1.6-89.2 months). We acknowledge that longer-term follow-up is needed to confirm curability in this population.

### Limitations

This study has some limitations. Although retrospective studies of data merged from different institutions have inherent limitations, a high degree of data quality assurance measures was instituted, including the creation of a standardized data dictionary (eMethods in the Supplement). In addition, data abstraction and review was methodical, taking place over 39 months (eFigure in the Supplement) to minimize missing or inaccurate data. These data predated the more consistent adoption of immunotherapy,^21^ and the potential synergy between immunotherapy and SBRT to enhance survival outcomes is not addressed in the present study.

## Conclusions

Phase 3 RCTs are needed to confirm the OS and PFS benefits of extracranial OM ablated with SBRT. However, the data in this study provide a benchmark of OM outcomes and identify meaningful factors associated with OS. Primary tumor type, a metachronous presentation more than 24 months since initial diagnosis, and the site of OM presentation can inform clinical decision-making and clinical trial design.

## Notes

Supplement. **eMethods.** Data Coding Instruction Book **eFigure.** Schematic Outlining Data Quality Assurance Process and Timeline **eTable 1.** Characteristics of Oligometastases Treated With SBRT **eTable 2.** Combinations of Anatomic Sites Treated With SBRT **eTable 3.** Survival Statistics **eTable 4.** Description of Adverse Events
