# Supplementary material for: Evaluation of Definitive Stereotactic Body Radiotherapy and Outcomes in Adults With Extracranial Oligometastasis
Source: JAMA Netw Open. 2020 Nov 16;3(11):e2026312. doi: 10.1001/jamanetworkopen.2020.26312 (PMC7670310; doi:10.1001/jamanetworkopen.2020.26312)
Supplement: Supplement. — eMethods. Data Coding Instruction Book eFigure. Schematic Outlining Data Quality Assurance Process and Timeline eTable 1. Characteristics of Oligometastases Treated With SBRT eTable 2. Combinations of Anatomic Sites Treated With SBRT eTable 3. Survival Statistics eTable 4. Description of Adverse Events [file jamanetwopen-e2026312-s001.pdf]

Supplementary Online Content

Poon I, Erler D, Dagan R, et al. Evaluation of definitive stereotactic body radiotherapy and outcomes in adults with extracranial oligometastasis. *JAMA Netw Open*. 2020;3(11):e2026312. doi:10.1001/jamanetworkopen.2020.26312

eMethods. Data Coding Instruction Book

eFigure. Schematic Outlining Data Quality Assurance Process and Timeline

eTable 1. Characteristics of Oligometastases Treated With SBRT

eTable 2. Combinations of Anatomic Sites Treated With SBRT

eTable 3. Survival Statistics

eTable 4. Description of Adverse Events

This supplementary material has been provided by the authors to give readers additional information about their work.

© 2020 Poon I. *JAMA Network Open*.

eMethods. Data Coding Instruction Book

The CORE database Guidebook outlines each form and gives details on the data fields that will be captured. It provides a description of the data fields, examples and comments to help explain the data that needs to be captured. For any numerical value that is N/A, please enter -999.

General Database Layout

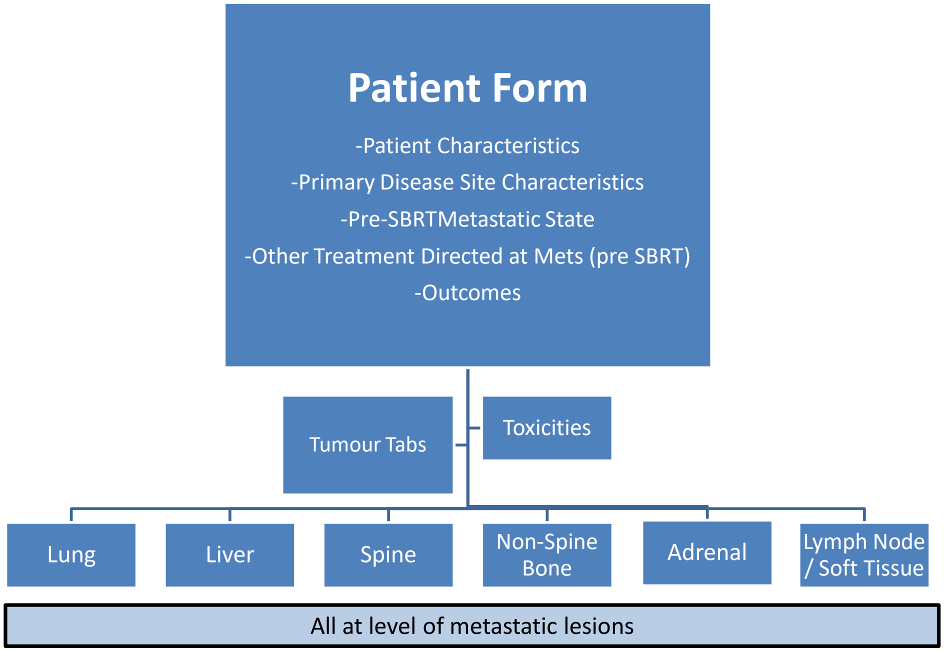

Below is a snap shot of the database. The tabs allow you to toggle between the forms to enter data.

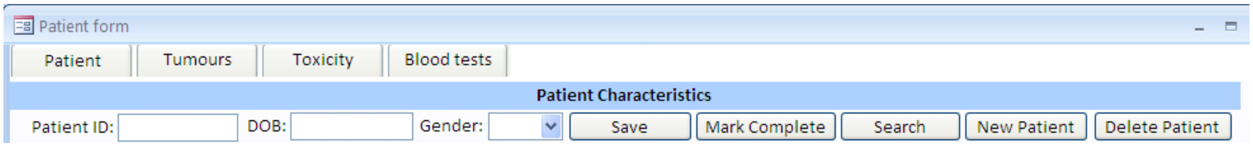

<u>Patient Form Instructions</u>

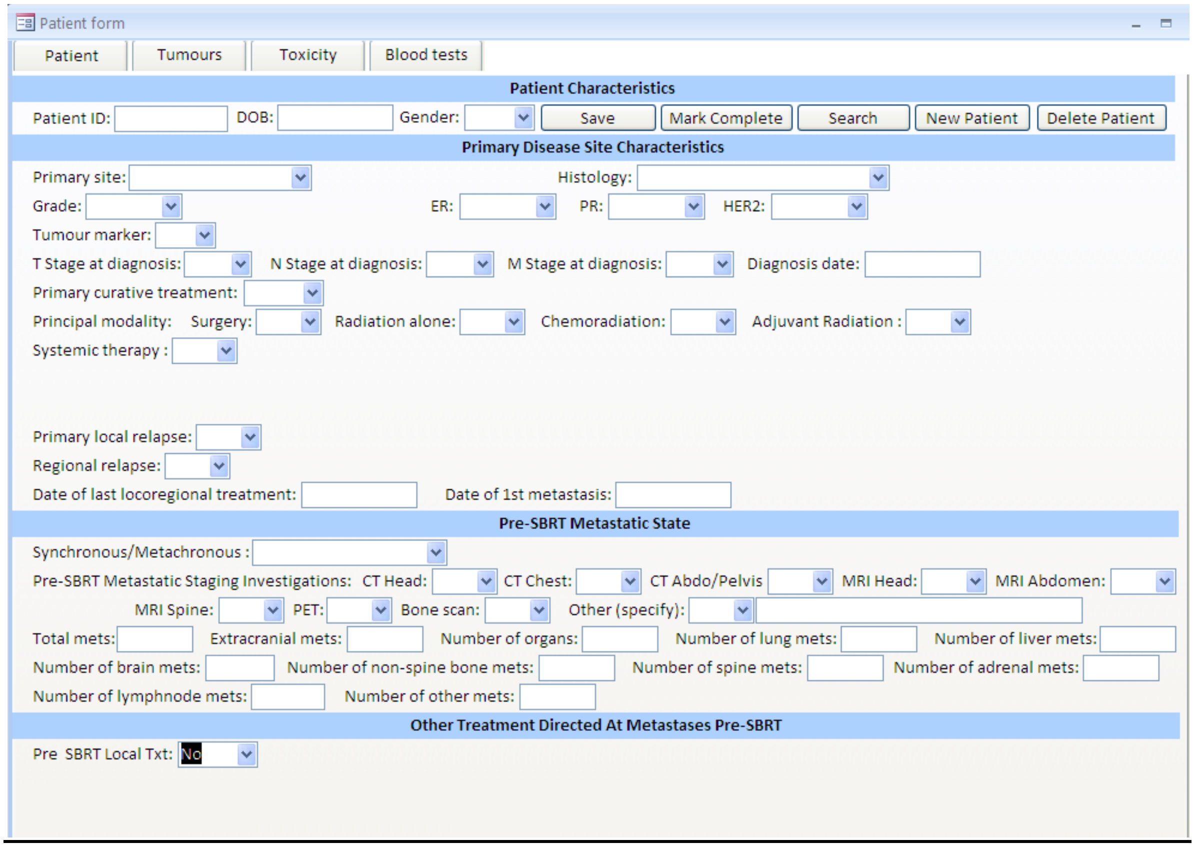

Patient form

Patient | Tumours | Toxicity | Blood tests

Patient Characteristics

Patient ID: DOB: Gender: Save Mark Complete Search New Patient Delete Patient

Primary Disease Site Characteristics

Primary site: Histology:
Grade: ER: PR: HER2:
Tumour marker:
T Stage at diagnosis: N Stage at diagnosis: M Stage at diagnosis: Diagnosis date:
Primary curative treatment:
Principal modality: Surgery: Radiation alone: Chemoradiation: Adjuvant Radiation :
Systemic therapy :

Primary local relapse:
Regional relapse:
Date of last locoregional treatment: Date of 1st metastasis:

Pre-SBRT Metastatic State

Synchronous/Metachronous :
Pre-SBRT Metastatic Staging Investigations: CT Head: CT Chest: CT Abdo/Pelvis MRI Head: MRI Abdomen:
MRI Spine: PET: Bone scan: Other (specify):
Total mets: Extracranial mets: Number of organs: Number of lung mets: Number of liver mets:
Number of brain mets: Number of non-spine bone mets: Number of spine mets: Number of adrenal mets:
Number of lymphnode mets: Number of other mets:

Other Treatment Directed At Metastases Pre-SBRT

Pre SBRT Local Txt: No

<u>PATIENT CHARACTERISTICS</u>

Variable	Description	Comments
Patient ID	Patient identification number	eg. 1-123 – first number will reflect the site that contributed the patient data as per their assigned number below and second number is a unique identifier for the patient at that site
DOB	Date of Birth	
Gender	Male/Female	

1. Sunnybrook Odette Cancer Centre start numbering at 1-001
2. University of Florida-J start numbering at 2-001
3. University of Florida-G start numbering at 3-001
4. Princess Alexandra Hospital start numbering at 4-001
5. Johns Hopkins University start numbering at 5-001
6. University Hospitals Case Medical Center start numbering at 6-001
7. University of Torino start numbering at 7-001

PRIMARY DISEASE SITE CHARACTERISTICS

Variable	Description	Comments
Primary Site	Select primary diagnosis from drop down list eg. Lung, breast, colon	Please select other and manually type in if diagnosis is not available
Histology	Select from drop down list eg. Adenocarcinoma	Please select other and manually type in if histology is not listed. Specify ductal or lobular for breast cancer histology.
Grade	Histologic grade (1-4, N/A)	This applies only to certain pathologies eg. breast, kidney. Please enter N/A if Grade is not specified.
Gleason Score	For prostate cancer only	Score from 2-10 based on pathology report. Should also be stated in clinical note.
Breast Cancer Details	ER, PR and HER2 receptor status	Histologic details eg. ER /PR/ HER2 status should be from primary pathology report even if biopsy was done on metastasis. Positive, negative or unknown.
Tumour Markers	If biopsy sample tested positive for molecular markers select, identify which receptors are positive	ALK, BRAF, Ckit, EGFR, Met Exon 14, KRAS, MSI, NRAS, RAS, RET, ROS-1
T Stage	At Diagnosis – select from drop down	Staging should be based on most recent version of AJCC<br><br>Enter pathological staging if possible and diagnosis date will be date of surgery.
N Stage	At Diagnosis – select from drop down	Enter pathological staging if possible and diagnosis date will be date of surgery.
M Stage	At Diagnosis – select from drop down	
Diagnosis Date	Date of primary cancer diagnosis	Should equal the date of biopsy or surgery and not the report date.
Primary Curative Treatment	Did the patient have curative treatment directed at primary disease? Y/N? If yes, enter the principal modality of primary curative treatment	This could occur chronologically after treatment of a metastasis eg. NSCLC patient who presents with brain met that is treated first and then SBRT is given to primary lung lesion.
Principal Modality of Primary Curative Treatment	Answer yes or no to all options Surgery (y/n), Radiation Alone (y/n), Chemoradiation (y/n),	May select more than 1 option. Sandwich treatment i.e. 3 cycles of chemotherapy, then radiation therapy followed by 3 more cycles of

	Adjuvant Radiation (y/n)	chemotherapy can be coded as chemoradiation
Systemic Therapy For Primary Disease	Identify if patient had systemic therapy for their primary cancer. If yes, identify the modality (can select more than 1 type)	If treated with targeted therapy, please identify subtype – can select more than one type of targeted therapy
Primary Local Relapse	Answer yes if there was a local recurrence of the primary disease site prior to development of metastases. Enter the method of salvage if applicable.	All primary local relapses must be reviewed by site investigator.<br><br>If local recurrence occurs after the patient is metastatic, it does not need to be entered
Regional Relapse	Answer yes if there was a regional recurrence of the primary disease before development of metastases and enter the method of salvage if applicable.	Eg. Melanoma - surgical excision of primary disease followed by nodal relapse months-years after<br>Must be reviewed by site investigator.
Date of Last Loco-regional Treatment	Record if regional treatment was given at a different date (usually later date) of initial local curative treatment or if patient had a local recurrences that was salvaged prior to presentation of first metastasis	For example above, date of radiation therapy or surgery to nodal relapse

Diagnosis Date (this logic applies to any date entered into the database)

- If information in chart is ambiguous eg. patient was diagnosed with prostate cancer in 2008 (only year of diagnosis is recorded), default to January 1st of the year and enter diagnosis date as Jan 1, 2008
- If month and year is recorded eg. patient was diagnosed with breast cancer in July 2010, default to July 1st and enter diagnosis date as July 1, 2010.

PRE-SBRT METASTASTIC STATE

Variable	Description	Comments
Date of 1st Metastasis	Date 1st metastasis discovered	Based on date of imaging that corresponds to clinical call of definite metastasis and not necessarily biopsy date
Synchronous / Metachronous	Synchronous /Metachronous drop down menu	Synchronous – patient presents with metastatic disease or metastatic disease is discovered within 6 months of diagnosis Metachronous - metastatic disease appears >6 months after treatment of primary
Metastatic Staging Investigations	Select yes for all applicable - CT head, CT chest, CT abdo/pelvis, MRI head, MRI abdomen, MRI spine, PET, Bone Scan, Other (specify)	List of staging investigations performed prior to (within 4 months) SBRT treatment for oligometastases
Total Mets	Total number of metastases at the time of first SBRT treatment for oligometastatic disease	For data collection purposes. Include brain metastases and metastases that have been treated with other modalities in this number. The highest number of brain metastases that should contribute to the count is 10. i.e. If report or clinical note states that the patient has innumerable brain metastases, this should equal 10.
Total Extracranial Mets	Total number of extracranial metastases at the time of first SBRT treatment for oligometastatic disease. Used for inclusion criteria.	This includes metastases that have been treated with other modalities and must be <6 to be included in the database. Extracranial is any metastasis located outside of the brain. Skull bone metastasis or base of brain bone metastasis are considered extracranial.
Number of Organs	Number of organs involved with metastases at the time of first SBRT treatment for oligometastatic disease.	Spine metastases are considered as a separate organ from non-spine bone metastases. Lymph nodes are considered as a single organ.
Number of Lung Mets, Liver Mets, Brain Mets, Spine Mets, Non Spine Mets, Adrenal Mets, Lymph Nodes, Other Mets (specify).	Number of metastases present in each category patient prior to SBRT for oligometastatic disease	Those that have been surgically resected or otherwise treated contribute to the count. Sacral metastases should be considered spine metastases. Each lymph node counts as a metastasis in overall count
PRE SBRT PSA	PSA value immediately before SBRT	Applies to prostate cancer patients only.
PRE SBRT CEA	CEA value immediately before SBRT	Applies to colorectal cancer patients only.

OTHER TREATMENT DIRECTED AT METASTASES PRE-SBRT

Goal of this section is to document any treatment delivered to sites of metastatic disease prior to SBRT,

Variable	Description	Comments
PRE SBRT METS TX	Was there treatment to a metastasis prior to SBRT? (Y/N)	If no, all known sites of disease should be answered and everything in this section should be n/a and section is closed
ALL KNOWN SITES OF DISEASE TREATED	At time of first SBRT for metastatic disease, were all known sites of disease treated with ablative/radical treatment? (Y/N)	Ablative/radical treatment = SBRT, surgery, RFA, radical conventional radiation therapy, not systemic therapy. Patients who have more than 1 site of metastases with no plan to treat (radically or ablatively) at time of initial SBRT should be excluded.
LUNG, LIVER, NON SPINE, SPINE, ADRENAL, LYMPH NODES, OTHER	Surgery, RFA, Conventional radiation therapy (xrt), , other (specify)	Indicate number of metastases that were treated with each modality for each anatomical category
PRE SBRT SYSTEMIC THERAPY	Did the patient receive systemic therapy for metastatic disease prior to the SBRT treatment?	Select each category of systemic therapy ie. cytotoxic chemotherapy, hormone therapy, immunotherapy, targeted therapy If treated with targeted therapy, please identify subtype(s)
PRE SBRT BRAIN METS	Did the patient present with brain metastases before having SBRT for oligometastatic disease? (Y/N)	If yes, indicate number of metastases (1, 2-5, 6-10, >10) innumerable = >10
PRE SBRT BRAIN MET TREATMENT	Identify how the brain metastases were treated as well as the number that was treated with each modality.	WBRT = whole brain radiotherapy, WB+SRS boost, SRS, none, other

OVERALL OUTCOMES

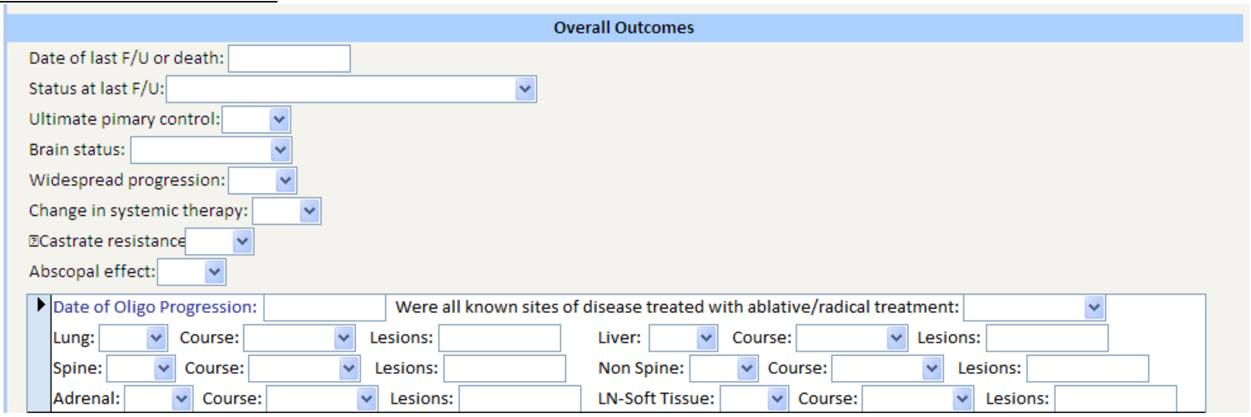

Variable	Description	Comments
DATE OF LAST F/U or DEATH	Date of last follow up or death	
STATUS AT LAST F/U	Alive (NED), Alive with OM Disease, Alive with widespread disease, Death with Disease, Death without Disease, Death from Toxicity (drop down)	OM = oligometastatic<br>Death without disease<br>Death from toxicity – please specify toxicity
ULTIMATE PRIMARY CONTROL	At time of last F/U or death, was the patient's primary site of disease under control? Y,N, unknown	
BRAIN STATUS	At time of last F/U or death what was the status of the patient's brain disease?	Select from Controlled, Uncontrolled (=active disease), Unknown, N/A<br>Select N/A for patients that have never had brain metastases
WIDESPREAD PROGRESSION	Does the patient have >6 sites of new extra cranial disease. If yes, enter date and use date of imaging that corresponds with clinician call. Y/N	Stop detailed follow-up after this date, except to determine date of death
CHANGE IN SYSTEMIC THERAPY	Is the patient starting new (i.e. Palliative) systemic therapy because of disease progression (that can't be treated with local therapy and not related to toxicities)? Y/N	If yes, enter the date and identify the systemic therapy modality that the patient will be started on.<br><br>Use date corresponding to note when clinician indicates that the change is going to occur
CASTRATE RESISTANCE	Is a patient who previously had hormone sensitive prostate cancer now castrate resistant? Date of castrate resistance corresponds to date of clinical note. Y/N	This question will appear only if a diagnosis of prostate cancer is selected. Patient is considered castrate resistant if they exhibit biochemical failure = rising PSA (>2 above nadir) or clinical progression on continuous (not intermittent) Androgen deprivation therapy (ADT)
ABSCOPAL EFFECT	Did the patient have imaging evidence of an abscopal effect? Y/N.	Abscopal effect occurs when there is evidence of spontaneous remission of metastases other than those that were treated with local ablative therapies in

		absence of systemic therapy including immunotherapy. All abscopal effects must be reviewed by the site investigator.<br>If yes, enter the date of imaging and enter details of the effect.
DATE OF OLIGO PROGRESSION #1, #2, #3, #4, #5	Date of distant recurrence after initial metastasis directed SBRT treatment shown on imaging that corresponds with clinician call	See below for definition of oligo progression. Include each site of oligo progression and the number of lesions for each site. After entry of oligo-progression #1, a new box will appear to populate should there be a second oligo-progression. If an oligo-progression is subsequently treated with SBRT, it can be linked to its course.<br>All oligoprogression events must be reviewed by the site investigator.
KNOWN SITES TREATED	Were all known sites of disease ablatively treated after oligoprogression?	To answer yes, all sites of oligoprogression must be treated with surgery, SBRT, RFA, radical XRT and not systemic therapy alone

Status at Last Follow Up

- Alive – the patient is alive and has no evidence of any active/untreated cancer
- Alive with OM Disease – the patient is alive but has evidence of <6 extracranial metastases that are active/untreated
- Alive with Widespread Disease – the patient is alive but has evidence of ≥6 extracranial metastases that are active/untreated
- Death with Disease - patient had active/uncontrolled cancer at the time of their death
- Death without Disease – patient had no active cancer at the time of their death
- Death from Toxicity – patient died as a result of a toxicity secondary to SBRT

Date of Oligoprogression

- Classified as oligoprogression when there are <6 new extracranial metastases evident at time of follow up
- Oligoprogression #1 is the first distant recurrence after SBRT – can have more than 1 site of recurrence for a single oligoprogression, Oligoprogression #2 is the second event of distant recurrence etc…..

© 2020 Poon I. *JAMA Network Open*.

LOCAL CONTROL - AFTER SBRT

- Local control will be recorded at the metastatic lesion level within the specific anatomic subsection

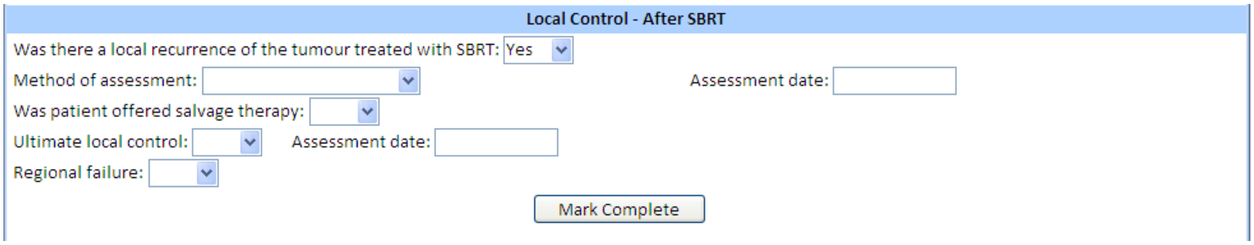

Variable	Description	Comments
LOCAL REC	Was there a local recurrence of the tumour treated with SBRT? (Y/N)	In clinical notes, it must specifically say that there has been a local recurrence. Suspicion of local recurrence is not sufficient.<br>All local recurrences must be reviewed by investigator on site
METHOD OF ASSESSMENT	How was local control evaluated? Select from Imaging-CT, Imaging-MRI, imaging-PET, Imaging-unknown, blood work, clinical exam	Imaging is preferred method of assessment and other options should be selected only in situations where the treated tumour was not imaged after SBRT
ASSESSMENT DATE	Imaging date that corresponds to clinician call of local recurrence or the most recent date of imaging in cases where there has not been a local recurrence	If blood test or clinical exam is the only method of assessment on record, please record the corresponding date. For patients that have not had a local recurrence, the assessment date should correspond to their last imaging study
SALVAGE	Was patient offered salvage therapy (Y/N) This question will only appear if there was a local recurrence	Salvage treatment = treatment to the area of the local recurrence ie. surgical resection, repeat SBRT, RFA
SALVAGE DETAILS	Select modality from drop down	Salvage chemotherapy should not be the same agent as preSBRT chemo and should involve a switch to next line post SBRT
ULTIMATE LOCAL CONTROL	If there was a local recurrence after SBRT, was local control achieved by salvage treatment?<br><br>Y/N/unknown	Should only appear if there was a local recurrence. Answer yes if the metastasis remained under control after salvage treatment. Date of assessment should be the imaging date closest to last F/U or death.
REGIONAL FAILURE	Applicable only to non-spine bone mets and LN/soft tissue tumour forms. Did patient have regional failure (Y/N)?	Regional failure = new met in same bone treated with SBRT that appears to be beyond the PTV or new node in same nodal chain as previously given SBRT. If yes, record date of failure and enter details in free text box<br><br>All regional failures must be reviewed by site investigator.

© 2020 Poon I. *JAMA Network Open*.

<u>Tumour Form Instructions</u>

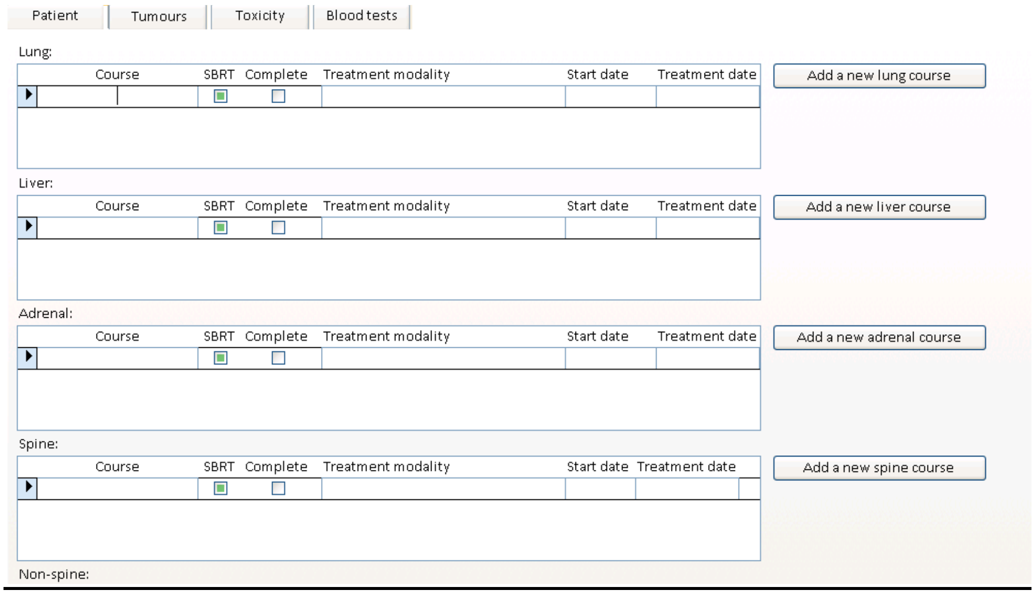

- This screen summarizes all metastatic tumours that are treated throughout a patient's oligometastatic trajectory starting with the first tumour that is treated with SBRT
- Any "non-ablative" treatment given to a metastasis that is eventually treated with SBRT should **not** be recorded as a separate tumour form ie. if a bone metastasis is first treated with conventional palliative radiation therapy and then is treated with SBRT, there should only be one tumour form entered and it should record the details of the SBRT
- If a course is entered in error, it can be deleted by clicking on the arrow on the left hand side to highlight it in black and hitting the delete button on your keyboard

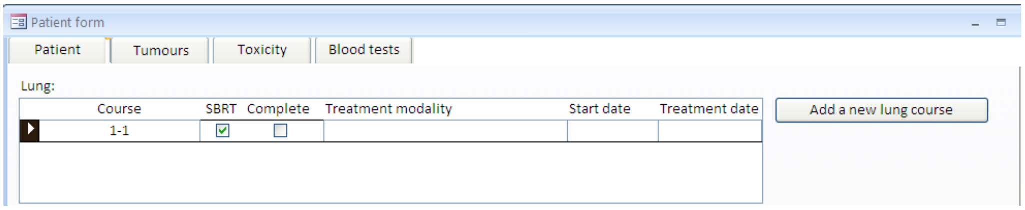

- You will then see this warning message and can select yes if you want to delete the course.

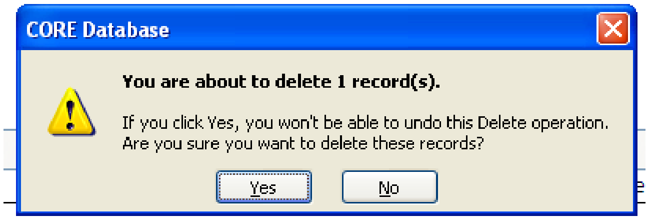

- The following instructions are for the forms created for each anatomical site

© 2020 Poon I. *JAMA Network Open*.

Lung Form Instructions

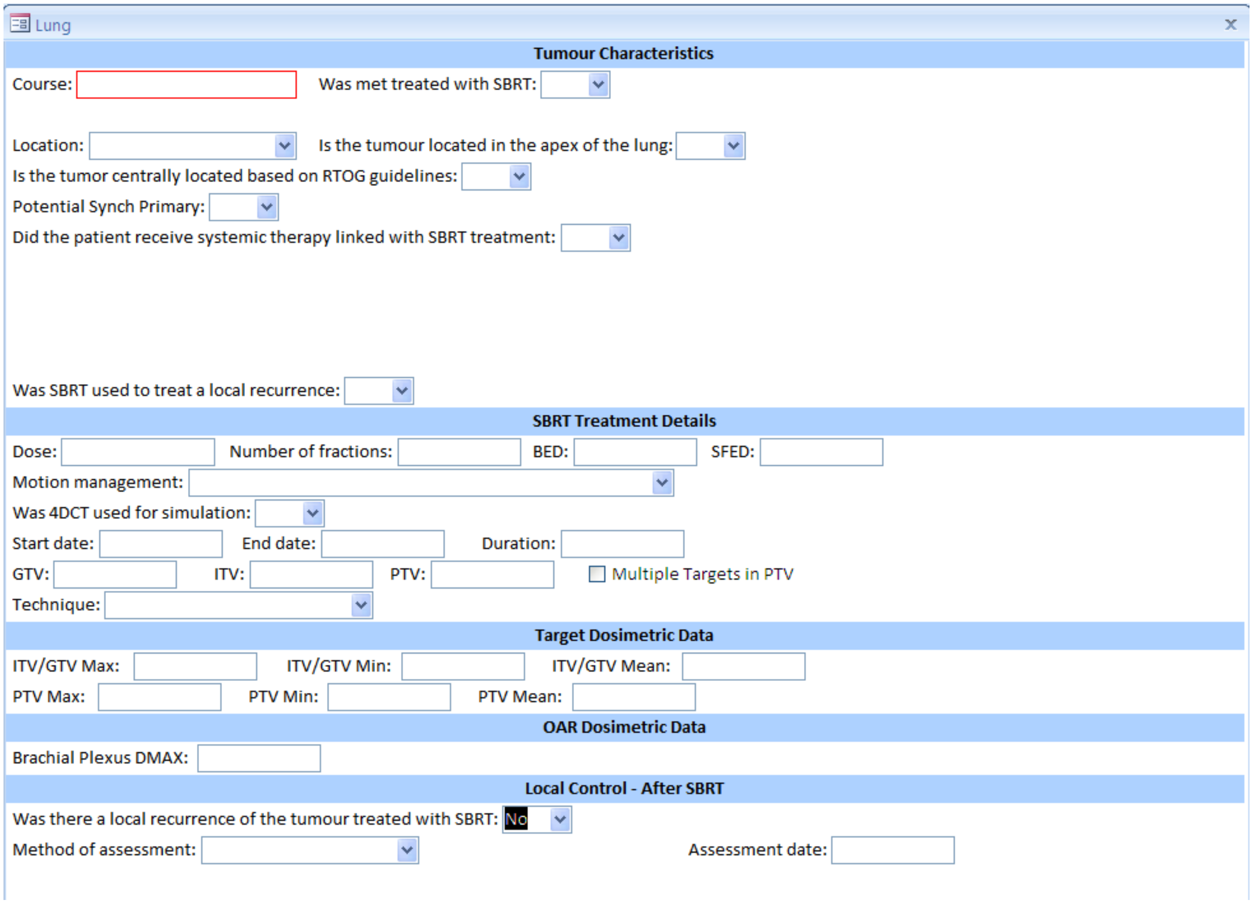
Lung

Tumour Characteristics

Course: Was met treated with SBRT:

Location: Is the tumour located in the apex of the lung:

Is the tumor centrally located based on RTOG guidelines:

Potential Synch Primary:

Did the patient receive systemic therapy linked with SBRT treatment:

Was SBRT used to treat a local recurrence:

SBRT Treatment Details

Dose: Number of fractions: BED: SFED:

Motion management:

Was 4DCT used for simulation:

Start date: End date: Duration:

GTV: ITV: PTV: Multiple Targets in PTV

Technique:

Target Dosimetric Data

ITV/GTV Max: ITV/GTV Min: ITV/GTV Mean:

PTV Max: PTV Min: PTV Mean:

OAR Dosimetric Data

Brachial Plexus DMAX:

Local Control - After SBRT

Was there a local recurrence of the tumour treated with SBRT: No

Method of assessment: Assessment date:

TUMOUR CHARACTERISTICS

Variable	Description	Comments
COURSE	A unique number assigned to each metastasis in order to follow it separately	eg. 2-4 first number represents the course number of SBRT and the second number represents the metastasis # (2nd course of SBRT but 4th metastasis). If 2 metastases are treated with a single plan, each metastasis must be assigned its own number to track local control and record specific dosimetric parameters.
SBRT	Was met treated with SBRT?(Y/N)	If no, indicate treatment modality, date of treatment and the course will be closed
LOCATION	Location of lung metastasis (drop down)	LUL, lingula, LLL, RUL, RML, RLL, not specified
APICAL	Is the tumour located in the apex of the lung?	Tumor considered apical when it is within 3 cm of the pleura in any direction from the apex (most superior slice) of the lung.
CENTRAL	Is the tumor centrally located based on RTOG guidelines (Y/N)	-see diagram and example below

© 2020 Poon I. *JAMA Network Open*.

POTENTIAL SYNCHRONOUS PRIMARY	Could the patient potentially have synchronous primary lung cancers instead of oligometastatic disease? (Y/N)	Any recurrence in the lung, regardless of primary treatment, as long as histology is not known to be different. Eg. Prev lobectomy for Stage 1 NSCLC and patient presents with a solitary lesion in contralateral lung 4 years later. Include as oligometatastic patient but flag that the patient could have synchronous primary lung cancers. There is no maximum time between presentations of tumours or maximum number of lung tumours.
SYSTEMIC THERAPY	Did the patient receive systemic therapy linked with SBRT treatment?	If yes, answer each: Pre-SBRT, concurrent, planned post and indicate modality See definition of each below
TYPE OF SYSTEMIC THERAPY	Identify type of systemic therapy (may select more than 1 option)	If targeted therapy is selected, please identify type(s)
SBRT FOR LOCAL RECURRENCE	Was SBRT used to treat a local recurrence of a previously treated met?	This should be explicit in clinical notes. Record previous failed mode of local treatment

Tumour Location

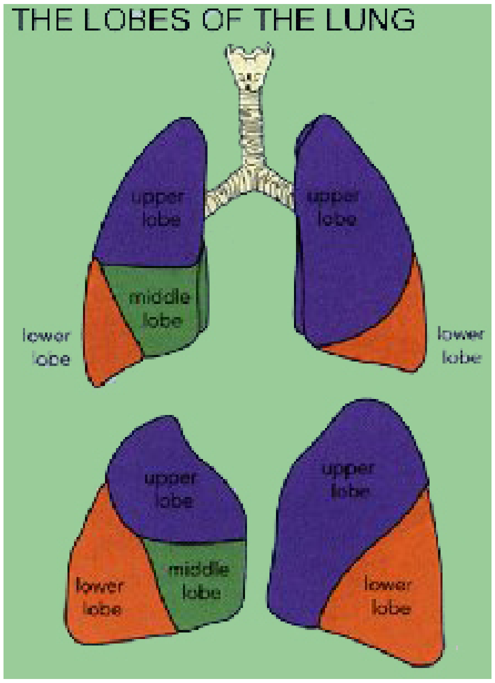

Tumours Considered Apical

© 2020 Poon I. *JAMA Network Open*.

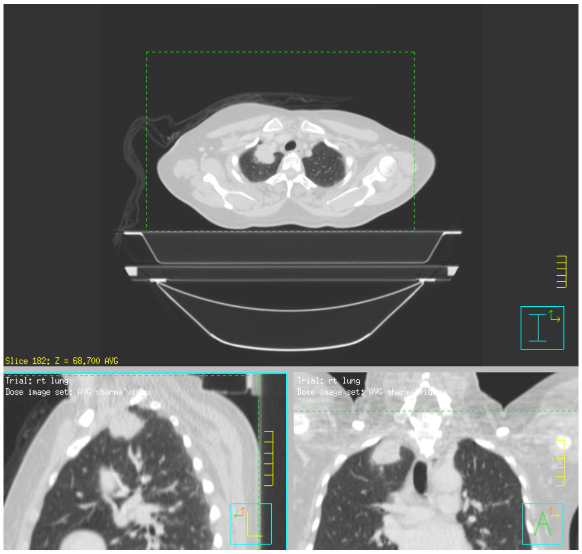

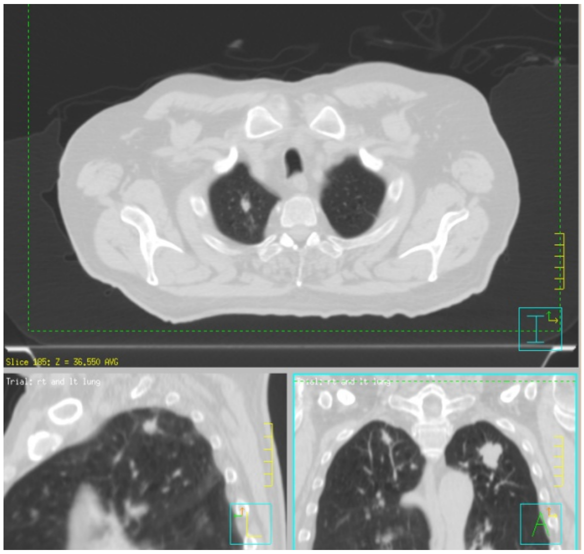

Central Tumours

- Central tumours are those touching any surface within 2 cm in all directions around the proximal bronchial tree (defined as carina, right and left main bronchi, right and left upper lobe bronchi, intermedius bronchus, right middle lobe bronchus, lingular bronchus right and left lower lobe bronchi)
- Tumors that are immediately adjacent to mediastinal or pericardial pleura (PTV touching the pleura) also are considered central tumors

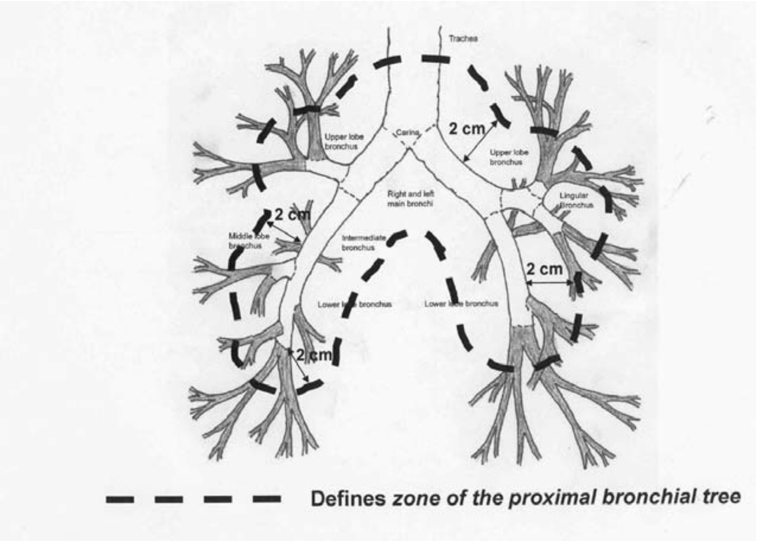

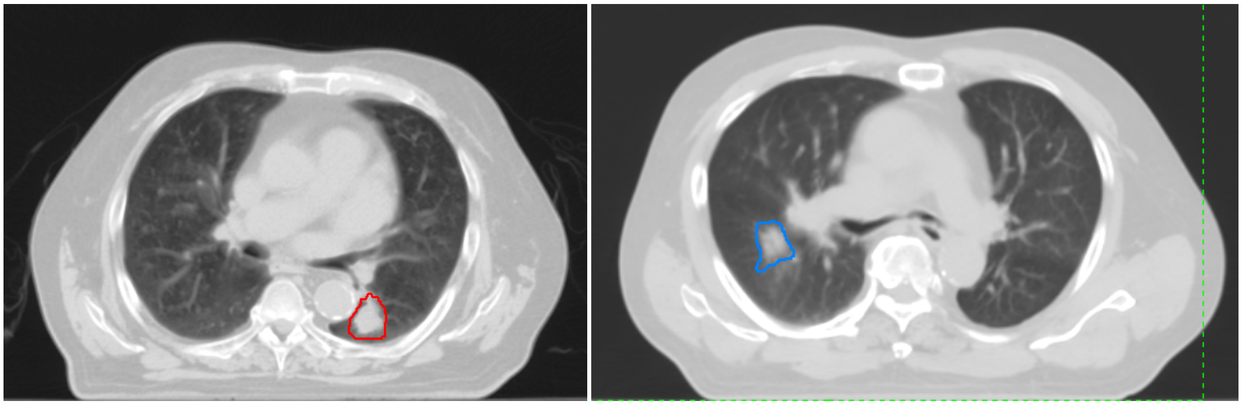

Systemic Therapy

- Pre-SBRT – Considered any systemic therapy that a patient is on immediately before SBRT. Example situations include when systemic therapy is given before SBRT in order to shrink the tumour to make it more amenable to treatment, or maintenance chemotherapy (eg. maintenance Pemetrexed in NSCLC) that is given before and after SBRT (should also be considered as Post-SBRT systemic) or targeted therapies that patients are on before and resume after SBRT. Select the time interval that corresponds to the break in systemic therapy prior to the start of SBRT: <1 week, 1-2 weeks, >2 weeks or unknown.
- Concurrent – the systemic agent is given during the course of SBRT – ie. there is no note of stopping the agent before the start of SBRT and remaining off for the duration of treatment or the SBRT is not intentionally scheduled between cycles of chemotherapy. If patients are on concurrent systemic therapy, please enter N/A in Pre-SBRT and Post-SBRT drop downs
- Post-SBRT – any systemic therapy that was planned to be given post SBRT –the decision to give chemotherapy is not based on response to treatment or a switch in agents based on response to treatment or appearance of new disease. Select the time interval that corresponds to the break in systemic therapy after completion of SBRT: <1 week, 1-2 weeks, >2 weeks or unknown.

SBRT TREATMENT DETAILS

Variable	Description	Comments
DOSE	Total dose prescribed (Gy)	Biological equivalent dose and single fraction equivalent dose will be calculated in spreadsheet using these values and assuming alpha/beta = 10
FX	Number of fractions	
MOTION MANAGEMENT	Indicate method of motion management: ABC, Abdominal Compression, Free Breathing/Nothing, Bodyfix, Gated, Other (specify) (drop down)	
4DCT	Was 4DCT used for simulation? (Y/N)	
START DATE	Date of Start of SBRT Treatment	
END DATE	Date of End of SBRT Treatment	Duration of the treatment will be calculated in the spreadsheet
GTV	Gross tumour volume (cc) measured on planning CT	Use the GTV for exhale phase if multiple GTVs are contoured. If 2

		lesions separate lesions make up the GTV, use half the volume for each entry.
ITV	Internal target volume (cc) if applicable (from planning CT)	If 2 lesions separate lesions make up the ITV, use half the volume for each entry.
PTV	Planning target volume (cc) (from planning CT)	If 2 lesions separate lesions make up the PTV, use half the volume for each entry.
MULTIPLE TARGETS IN PTV	Y/N – more than one tumour is contoured as ITV/PTV	
TECHNIQUE	Treatment delivery technique 3D, conformal arc, IMRT, VMAT, Cyberknife, other (specify) (drop down)	

TARGET DOSIMETRIC DATA

Variable	Description	Comments
ITV/GTV MAX	Maximum dose to ITV (Gy) (GTV if ITV not available)	Report dose to GTV only if ITV was not used.
ITV/GTV MIN	Minimum dose to ITV (GTV if ITV not available) (Gy)	
ITV/GTV MEAN	Mean dose to ITV (GTV if ITV not available) (Gy)	
PTV MAX	Maximum dose to PTV (Gy)	
PTV MIN	Minimum dose to PTV (Gy)	
PTV MEAN	Mean dose to PTV (Gy)	

- If possible, tabular DVH data for PTV should be uploaded and relabeled (see instructions at the end of the guide)

OAR DOSIMETRIC DATA

Variable	Description	Comments
BRACHIAL PLEXUS DMAX	Maximum point dose to brachial plexus (Gy)	Enter -999 if brachial plexus dose was not calculated or not available

© 2020 Poon I. *JAMA Network Open*.

<u>Liver Form Instructions</u>

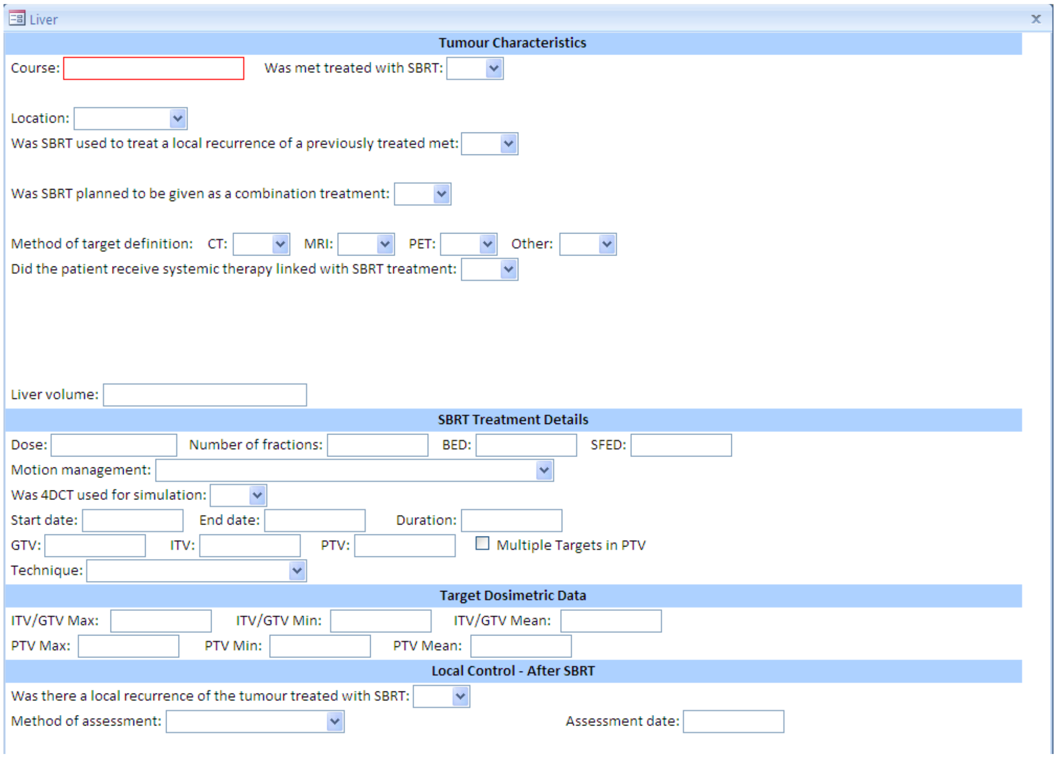

Liver

Tumour Characteristics

Course: | Was met treated with SBRT:

Location:

Was SBRT used to treat a local recurrence of a previously treated met:

Was SBRT planned to be given as a combination treatment:

Method of target definition: CT: | MRI: | PET: | Other:

Did the patient receive systemic therapy linked with SBRT treatment:

Liver volume:

SBRT Treatment Details

Dose: | Number of fractions: | BED: | SFED:

Motion management:

Was 4DCT used for simulation:

Start date: | End date: | Duration:

GTV: | ITV: | PTV: | Multiple Targets in PTV

Technique:

Target Dosimetric Data

ITV/GTV Max: | ITV/GTV Min: | ITV/GTV Mean:

PTV Max: | PTV Min: | PTV Mean:

Local Control - After SBRT

Was there a local recurrence of the tumour treated with SBRT:

Method of assessment: | Assessment date:

<u>TUMOUR CHARACTERISTICS</u>

Variable	Description	Comments
COURSE	Number assigned to track each metastasis	eg. 2-4 first number represents the course number of SBRT and the second number represents the metastasis # (2nd course of SBRT but 4th metastasis)
SBRT	Was met treated with SBRT?(Y/N)	If no, indicate treatment modality, date of treatment and the course will be closed
LOCATION	Central, peripheral, unknown	Tumour is considered central when any part of PTV overlaps with hilum or the first branch of the vessels after entering the porta (see examples below)
SBRT for LOCAL RECURRENCE –	Was SBRT used to treat a local recurrence of a previously treated met?	This should be explicit in clinical notes. Record previous failed mode of local treatment
COMBINATION TREATMENT	Was SBRT planned to be given as a combination treatment to a liver metastasis?	Should be explicitly stated in clinical note. If yes, record the treatment modality that it was combined with eg . SURGERY, RFA, TACE,,etc
METHOD OF TARGET DEFINITION	CT, MRI, PET, other (specify)	Imaging modalities used to define GTV/ITV for treatment plan. Select all that apply.
SYSTEMIC THERAPY	Did the patient receive systemic	If yes, answer each: Pre-SBRT,

	therapy linked with SBRT treatment?	concurrent, planned post and indicate modality. See explanation of each below
TYPE OF SYSTEMIC THERAPY	Identify type of systemic therapy (may select more than 1 option)	If targeted therapy is selected, please identify type(s)
LIVER VOLUME	Total liver volume (cc) as measured on planning CT scan	If 4DCT was used for planning, use liver volume measured from contour on exhale dataset

<u>Liver Location – Examples of Central tumours</u>

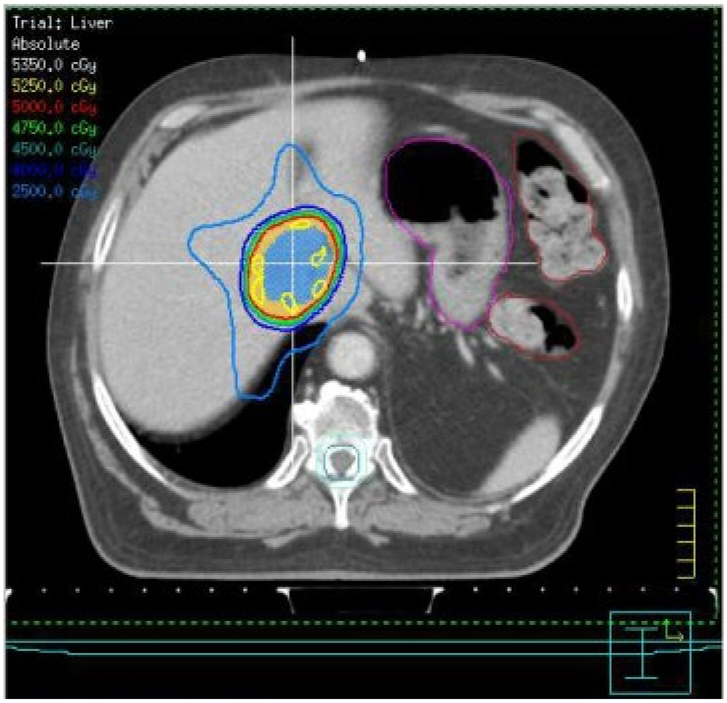

<u>Systemic Therapy</u>

- Pre-SBRT – Considered any systemic therapy that a patient is on immediately before SBRT. Example situations include when systemic therapy is given before SBRT in order to shrink the tumour to make it more amenable to treatment, or maintenance chemotherapy (eg. maintenance Pemetrexed in NSCLC) that is given before and after SBRT (should also be considered as Post-SBRT systemic) or targeted therapies that patients are on before and resume after SBRT. Select the time interval that corresponds to the break in systemic therapy prior to the start of SBRT: <1 week, 1-2 weeks, >2 weeks or unknown.
- Concurrent – the systemic agent is given during the course of SBRT – ie. there is no note of stopping the agent before the start of SBRT and remaining off for the duration of treatment or the SBRT is not intentionally scheduled between cycles of chemotherapy. If patients are on concurrent systemic therapy, please enter N/A in Pre-SBRT and Post-SBRT drop downs
- Post-SBRT – any systemic therapy that was planned to be given post SBRT –the decision to give chemotherapy is not based on response to treatment or a switch in agents based on response to treatment or appearance of new disease. Select the time interval that corresponds to the break in systemic therapy after completion of SBRT: <1 week, 1-2 weeks, >2 weeks or unknown.

© 2020 Poon I. *JAMA Network Open*.

SBRT TREATMENT DETAILS

Variable	Description	Comments
DOSE	Total dose prescribed (Gy)	Biological equivalent dose and single fraction equivalent dose will be calculated in spreadsheet using these values and assuming alpha/beta = 10
FX	Number of fractions	
MOTION MANAGEMENT	Indicate method of motion management: ABC, Abdominal Compression, Free Breathing/Nothing, Bodyfix, Gated, Other (specify) (drop down)	
4DCT	Was 4DCT used for simulation? (Y/N)	
START DATE	Date of Start of SBRT Treatment	
END DATE	Date of End of SBRT Treatment	Duration of the treatment will be calculated in the spreadsheet
GTV	Gross tumour volume (cc) measured on planning CT	Use the GTV for exhale phase if multiple GTVs are contoured.
ITV	Internal target volume (cc) if applicable (from planning CT)	
PTV	Planning target volume (cc) (from planning CT)	
MULTIPLE TARGETS IN PTV	Y/N – more than one tumour is contoured as ITV/PTV	
TECHNIQUE	Treatment delivery technique 3D, conformal arc, IMRT, VMAT, Cyberknife, other (specify) (drop down)	

TARGET DOSIMETRIC DATA

Variable	Description	Comments
ITV/GTV MAX	Maximum dose to ITV (Gy) (GTV if ITV not available)	Report dose to GTV only if there is no ITV used.
ITV/GTV MIN	Minimum dose to ITV (GTV if ITV not available)	
ITV/GTV MEAN	Mean dose to ITV (GTV if ITV not available)	
PTV MAX	Maximum dose to PTV	
PTV MIN	Minimum dose to PTV	
PTV MEAN	Mean dose to PTV	

- If possible, tabular DVH data for PTV should be uploaded and relabeled (see instructions at the end of the guide)

Adrenal Form Instructions

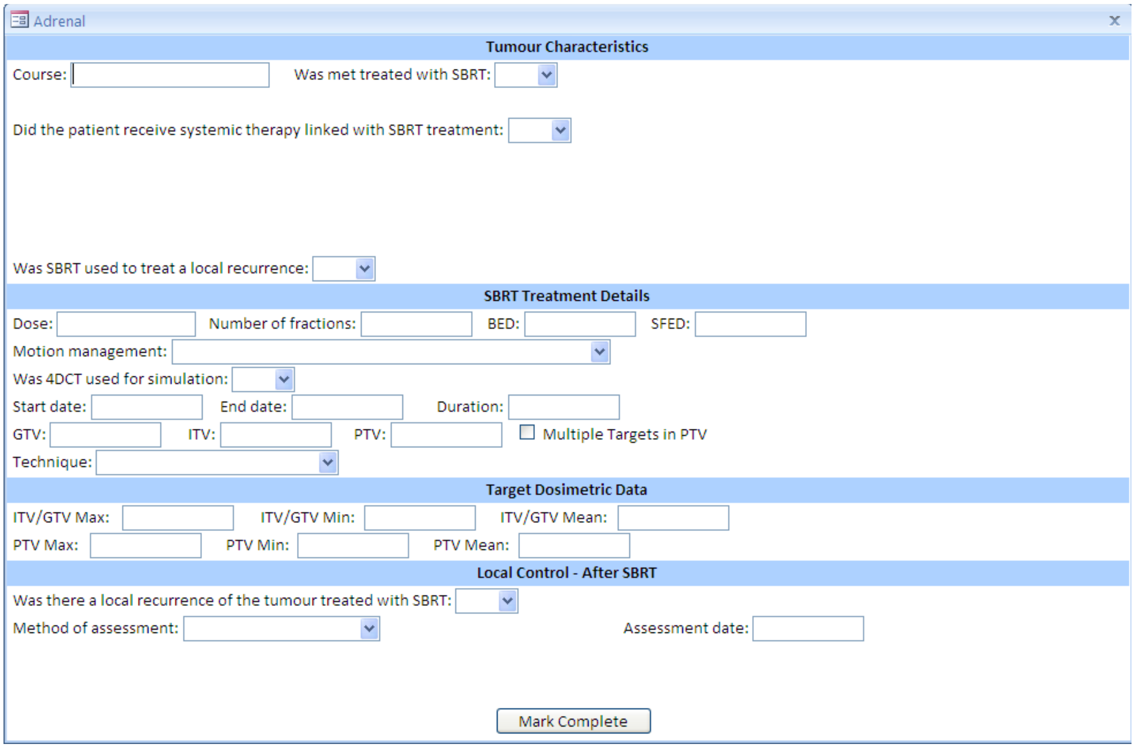
Adrenal

Tumour Characteristics

Course: Was met treated with SBRT:

Did the patient receive systemic therapy linked with SBRT treatment:

Was SBRT used to treat a local recurrence:

SBRT Treatment Details

Dose: Number of fractions: BED: SFED:

Motion management:

Was 4DCT used for simulation:

Start date: End date: Duration:

GTV: ITV: PTV: Multiple Targets in PTV

Technique:

Target Dosimetric Data

ITV/GTV Max: ITV/GTV Min: ITV/GTV Mean:

PTV Max: PTV Min: PTV Mean:

Local Control - After SBRT

Was there a local recurrence of the tumour treated with SBRT:

Method of assessment: Assessment date:

Mark Complete

TUMOUR CHARACTERISTICS

Variable	Description	Comments
COURSE	Number assigned to track each metastasis	eg. 2-4 first number represents the course number of SBRT and the second number represents the metastasis # (2nd course of SBRT but 4th metastasis)
SBRT	Was met treated with SBRT?(Y/N)	If no, indicate treatment modality, date of treatment and the course will be closed
SYSTEMIC THERAPY	Did the patient receive systemic therapy linked with SBRT treatment?	If yes, answer each: Pre-SBRT, concurrent, planned post and indicate modality. Se explanation of each below.
TYPE OF SYSTEMIC THERAPY	Identify type of systemic therapy (may select more than 1 option)	If targeted therapy is selected, please identify type(s)
SBRT for LOCAL RECURRENCE – add to each tumour form	Was SBRT used to treat a local recurrence of a previously treated met?	This should be explicit in clinical notes. Record previous failed mode of local treatment

Systemic Therapy

- Pre-SBRT – Considered any systemic therapy that a patient is on immediately before SBRT. Example situations include when systemic therapy is given before SBRT in order to shrink the tumour to make it more amenable to treatment, or maintenance chemotherapy (eg. maintenance Pemetrexed in NSCLC) that is given before and after SBRT (should also be considered as Post-SBRT systemic) or targeted therapies that patients are on before and resume after SBRT. Select the time interval that corresponds to the break in systemic therapy prior to the start of SBRT: <1 week, 1-2 weeks, >2 weeks or unknown.
- Concurrent – the systemic agent is given during the course of SBRT – ie. there is no note of stopping the agent before the start of SBRT and remaining off for the duration of treatment or the SBRT is not intentionally scheduled between cycles of chemotherapy. If patients are on concurrent systemic therapy, please enter N/A in Pre-SBRT and Post-SBRT drop downs
- Post-SBRT – any systemic therapy that was planned to be given post SBRT –the decision to give chemotherapy is not based on response to treatment or a switch in agents based on response to treatment or appearance of new disease. Select the time interval that corresponds to the break in systemic therapy after completion of SBRT: <1 week, 1-2 weeks, >2 weeks or unknown.

SBRT TREATMENT DETAILS

Variable	Description	Comments
DOSE	Total dose prescribed (Gy)	Biological equivalent dose and single fraction equivalent dose will be calculated in spreadsheet using these values and assuming alpha/beta = 10
FX	Number of fractions	
MOTION MANAGEMENT	Indicate method of motion management: ABC, Abdominal Compression, Free Breathing/Nothing, Bodyfix, Gated, Other (specify) (drop down)	
4DCT	Was 4DCT used for simulation? (Y/N)	
START DATE	Date of Start of SBRT Treatment	
END DATE	Date of End of SBRT Treatment	Duration of the treatment will be calculated in the spreadsheet
GTV	Gross tumour volume (cc) measured on planning CT	Use the GTV for exhale phase if multiple GTVs are contoured.
ITV	Internal target volume (cc) if applicable (from planning CT)	
PTV	Planning target volume (cc) (from planning CT)	
MULTIPLE TARGETS IN PTV	Y/N – more than one tumour is contoured as ITV/PTV	If 2 lesions separate lesions make up the PTV, use half the volume for each entry.
TECHNIQUE	Treatment delivery technique 3D, conformal arc, IMRT, VMAT, Cyberknife, other (specify) (drop down)	

© 2020 Poon I. *JAMA Network Open*.

TARGET DOSIMETRIC DATA

Variable	Description	Comments
ITV/GTV MAX	Maximum dose to ITV (Gy) (GTV if ITV not available)	Report dose to GTV only if there is no ITV used.
ITV/GTV MIN	Minimum dose to ITV (GTV if ITV not available)	
ITV/GTV MEAN	Mean dose to ITV (GTV if ITV not available)	
PTV MAX	Maximum dose to PTV	
PTV MIN	Minimum dose to PTV	
PTV MEAN	Mean dose to PTV	

- If possible, tabular DVH data for PTV should be uploaded and relabeled (see instructions at the end of the guide)

© 2020 Poon I. *JAMA Network Open*.

Spine Form Instructions

If treatment volume includes more than 1 vertebral level, it should be entered as a single course only if the disease is continuous and multiple vertebral levels are involved through paraspinal extension. Otherwise, each vertebra should be entered as a separate course/tumour in order to track local control. Eg. If treatment volume includes T6, T7, it can be entered as a single course if it is a single metastasis that spans both levels. If there are 2 discrete bone metastases, 1 on T6 and 1 on T7, they should each be entered as their own course.

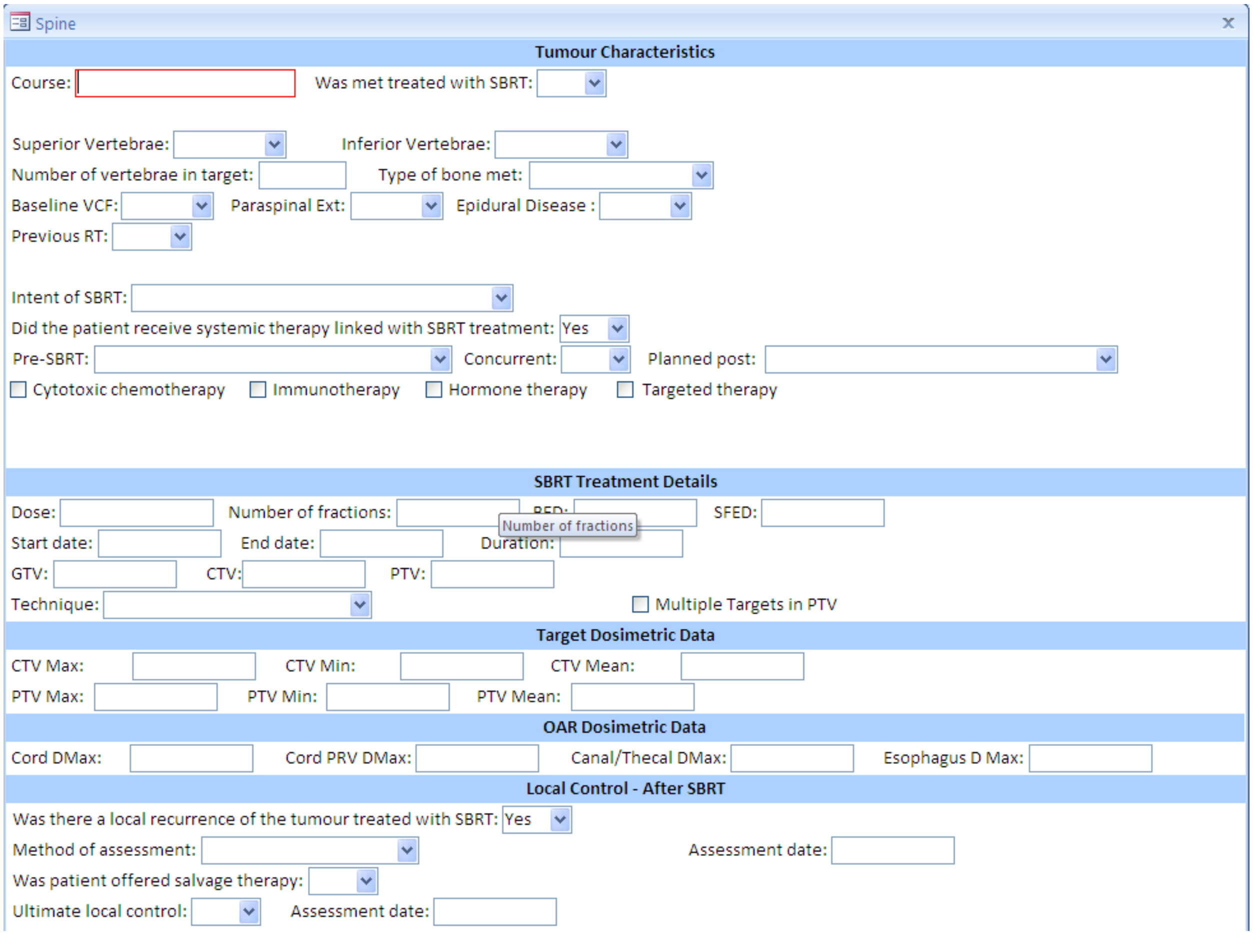

Spine

Tumour Characteristics

Course: Was met treated with SBRT:

Superior Vertebrae: Inferior Vertebrae:

Number of vertebrae in target: Type of bone met:

Baseline VCF: Paraspinal Ext: Epidural Disease :

Previous RT:

Intent of SBRT:

Did the patient receive systemic therapy linked with SBRT treatment: Yes

Pre-SBRT: Concurrent: Planned post:

☐ Cytotoxic chemotherapy ☐ Immunotherapy ☐ Hormone therapy ☐ Targeted therapy

SBRT Treatment Details

Dose: Number of fractions: BED: SFED:

Number of fractions

Start date: End date: Duration:

GTV: CTV: PTV:

Technique: ☐ Multiple Targets in PTV

Target Dosimetric Data

CTV Max: CTV Min: CTV Mean:

PTV Max: PTV Min: PTV Mean:

OAR Dosimetric Data

Cord DMax: Cord PRV DMax: Canal/Thecal DMax: Esophagus D Max:

Local Control - After SBRT

Was there a local recurrence of the tumour treated with SBRT: Yes

Method of assessment: Assessment date:

Was patient offered salvage therapy:

Ultimate local control: Assessment date:

© 2020 Poon I. *JAMA Network Open*.

TUMOUR CHARACTERISTICS

Variable	Description	Comments
COURSE	Number assigned to track each metastasis	eg. 2-4 first number represents the course number of SBRT and the second number represents the metastasis # (2nd course of SBRT but 4th metastasis)
SBRT	Was met treated with SBRT?(Y/N)	If no, indicate treatment modality, date of treatment and the course will be closed
SUPERIOR VERTEBRA	Select most superior vertebra in treatment volume.	If SBRT is given to single vertebra, superior and inferior should be the same vertebra.
INFERIOR VERTEBRA	Select most inferior vertebra in treatment volume.	
# VERTEBRAE IN TARGET	Number of vertebrae in SBRT target	
TYPE OF BONE MET	Type of bone metastasis: Lytic, Sclerotic, Mixed, N/A (drop down)	Sclerotic may also be referred to as blastic
BASELINE VCF	Was there evidence of vertebral compression fracture in target before SBRT?	See below for example of VCF. Baseline VCF should be explicitly commented on by clinician or in radiology report.
PARASPINAL EXT	Was there paraspinal extension of the target lesion?	Soft tissue disease extending outside of vertebrae. Will be commented on in radiology report. See below for example of paraspinal extension
EPIDURAL DISEASE	Was there epidural disease present?	Disease extending into spinal canal. Confirmed on MRI.
PREV RT	Was the spine met previously treated with radiation therapy?	If so, record date, dose and number of fractions that were delivered.
2nd Course of Prev RT	Was the target spine met irradiated a second time before SBRT?	If so, record date, dose and number of fractions that were delivered for the second course of radiation therapy.
INTENT OF SBRT	Reirradiation post op, de novo treatment (drop down)	Reirradiation only refers to previous radiation therapy directed at the same spine met
SYSTEMIC THERAPY	Did the patient receive systemic therapy linked with SBRT treatment?	If yes, answer each: Pre-SBRT, concurrent, planned post and indicate modality. See explanation of each below.
TYPE OF SYSTEMIC THERAPY	Identify type of systemic therapy (may select more than 1 option)	If targeted therapy is selected, please identify type(s)

Baseline Vertebral Compression

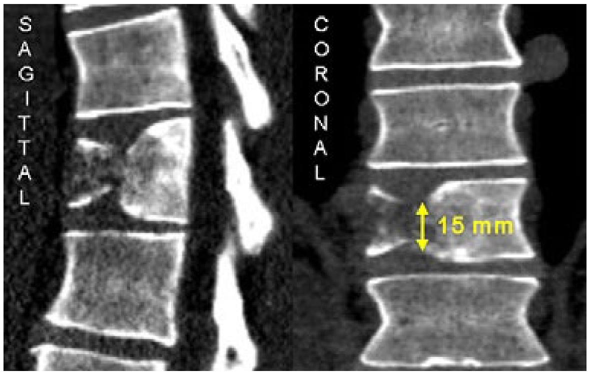

Image adapted from: Cunha M, Al-Omair A, Atenafu E, Masucci GL, Letourneau D, Korol R, Yu E, Howard P, Lochray F, da Costa L, Fehlings MG, Sahgal A. Vertebral Compression Fracture (VCF) After Spine Stereotactic Body Radiation Therapy (SBRT): Analysis of Predictive Factors. Int J of Radiat Oncol Biol Phys. 2012; 84(3):e343-349

Paraspinal Extension

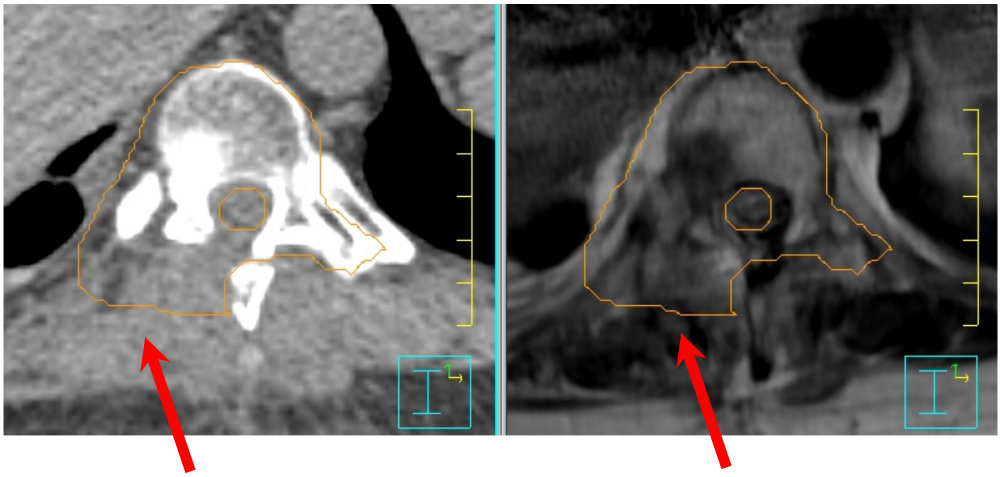

Epidural Disease

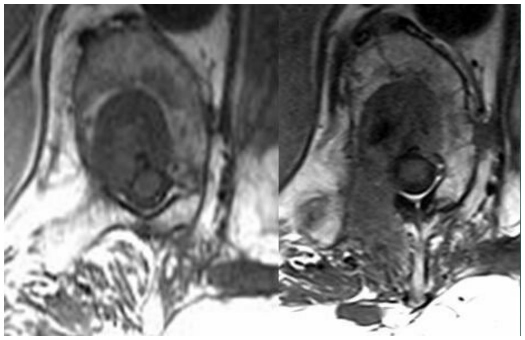

© 2020 Poon I. *JAMA Network Open*.

Systemic Therapy

- Pre-SBRT – Considered any systemic therapy that a patient is on immediately before SBRT. Example situations include when systemic therapy is given before SBRT in order to shrink the tumour to make it more amenable to treatment, or maintenance chemotherapy (eg. maintenance Pemetrexed in NSCLC) that is given before and after SBRT (should also be considered as Post-SBRT systemic) or targeted therapies that patients are on before and resume after SBRT. Select the time interval that corresponds to the break in systemic therapy prior to the start of SBRT: <1 week, 1-2 weeks, >2 weeks or unknown.
- Concurrent – the systemic agent is given during the course of SBRT – ie. there is no note of stopping the agent before the start of SBRT and remaining off for the duration of treatment or the SBRT is not intentionally scheduled between cycles of chemotherapy. If patients are on concurrent systemic therapy, please enter N/A in Pre-SBRT and Post-SBRT drop downs
- Post-SBRT – any systemic therapy that was planned to be given post SBRT –the decision to give chemotherapy is not based on response to treatment or a switch in agents based on response to treatment or appearance of new disease. Select the time interval that corresponds to the break in systemic therapy after completion of SBRT: <1 week, 1-2 weeks, >2 weeks or unknown.

SBRT TREATMENT DETAILS

Variable	Description	Comments
DOSE	Total dose prescribed (Gy)	Biological equivalent dose and single fraction equivalent dose will be calculated in spreadsheet using these values and assuming alpha/beta = 10
FX	Number of fractions	
START DATE	Date of Start of SBRT Treatment	
END DATE	Date of End of SBRT Treatment	Duration of the treatment will be calculated in the spreadsheet
GTV	Gross tumour volume (cc) measured from treatement plan (if applicable)	Report GTV only if directed to do so at your clinical site.
CTV	Clinical target volume (cc) if applicable	If 2 lesions separate lesions make up the CTV, use half the volume for each entry.
PTV	Planning target volume (cc) (from planning CT)	
MULTIPLE TARGETS IN PTV	Y/N – more than one tumour is contoured as CTV/PTV	If 2 lesions separate lesions make up the PTV, use half the volume for each entry.
TECHNIQUE	Treatment delivery technique 3D, conformal arc, IMRT, VMAT, Cyberknife, other (specify) (drop down)	

TARGET DOSIMETRIC DATA

Variable	Description	Comments
CTV MAX	Maximum dose to CTV (Gy)	
CTV MIN	Minimum dose to CTV	
CTV MEAN	Mean dose to CTV	
PTV MAX	Maximum dose to PTV	
PTV MIN	Minimum dose to PTV	
PTV MEAN	Mean dose to PTV	

- If possible, tabular DVH data for PTV should be uploaded and relabeled (see instructions at the end of the guide)

OAR DOSIMETRIC DATA

Variable	Description	Comments
CORD DMAX	Maximum point dose to spinal cord (Gy)	Record if readily available, enter 999 if not available
CORD PRV DMAX	Maximum point dose to cord PRV (Gy) (if applicable)	Record if readily available, enter 999 if not available<br>May also be named cord + 1.5 mm or cord + 2
CANAL/THECAL DMAX	Maximum point dose to spinal canal / thecal sac	Record if readily available, enter 999 if not available<br>May also be named cord + 5 mm
ESOPHAGUS DMAX	Maximum point dose to esophagus	Record if readily available, enter 999 if not available

Non-Spine Form Instructions

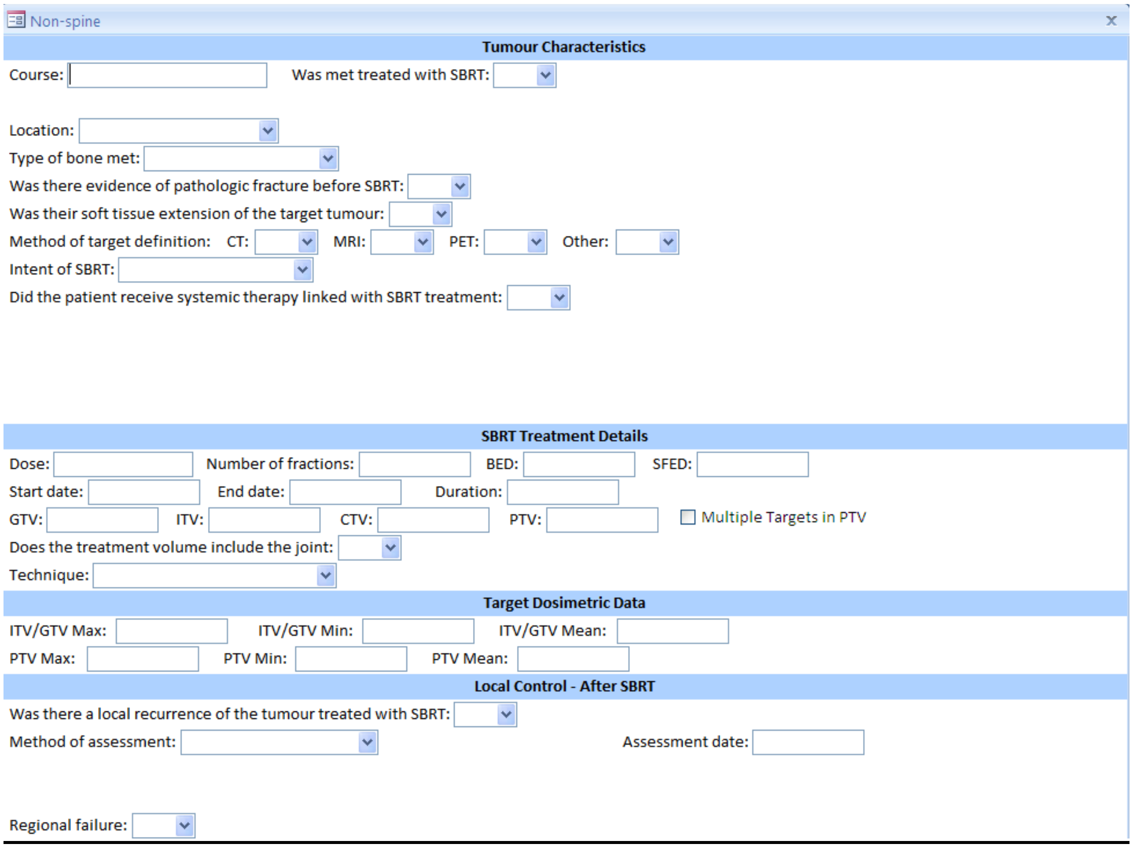
Non-spine

Tumour Characteristics

Course: ___ Was met treated with SBRT: ___

Location: ___

Type of bone met: ___

Was there evidence of pathologic fracture before SBRT: ___

Was their soft tissue extension of the target tumour: ___

Method of target definition: CT: ___ MRI: ___ PET: ___ Other: ___

Intent of SBRT: ___

Did the patient receive systemic therapy linked with SBRT treatment: ___

SBRT Treatment Details

Dose: ___ Number of fractions: ___ BED: ___ SFED: ___

Start date: ___ End date: ___ Duration: ___

GTV: ___ ITV: ___ CTV: ___ PTV: ___ ☐ Multiple Targets in PTV

Does the treatment volume include the joint: ___

Technique: ___

Target Dosimetric Data

ITV/GTV Max: ___ ITV/GTV Min: ___ ITV/GTV Mean: ___

PTV Max: ___ PTV Min: ___ PTV Mean: ___

Local Control - After SBRT

Was there a local recurrence of the tumour treated with SBRT: ___

Method of assessment: ___ Assessment date: ___

Regional failure: ___

TUMOUR CHARACTERISTICS

Variable	Description	Comments
COURSE	Number assigned to track each metastasis	eg. 2-4 first number represents the course number of SBRT and the second number represents the metastasis # (2nd course of SBRT but 4th metastasis)
SBRT	Was met treated with SBRT?(Y/N)	If no, indicate treatment modality and the course will be closed
LOCATION	Rib, Shoulder Girdle, Sternum, Upper Limb, Lower Limb, symphysis pubis, ischium, acetabulum, pelvic ala , Skull, Other (specify) (drop down)	Shoulder girdle = glenoid, clavicle, scapula
TYPE OF BONE MET	Type of bone metastasis: Lytic, Sclerotic, Mixed, N/A (drop down)	Sclerotic may also be referred to as blastic
BASELINE PATHOLOGIC FRACTURE	Was there evidence of pathologic fracture in target before SBRT?	Baseline pathologic fracture should be noted by clinician prior to start of treatment or in pre-treatment radiology report
SOFT TISSUE EXTENSION	Was their soft tissue extension of the target tumour?	See below for example of soft tissue extension

METHOD OF TARGET DEFINITION	CT, MRI, PET, other (specify)	Imaging modalities used to define GTV/ITV for treatment plan. Select all that apply.
INTENT OF SBRT	Reirradiation, post op, de novo treatment (drop down)	Reirradiation only refers to previous radiation therapy directed at a bone met in the same region
SYSTEMIC THERAPY	Did the patient receive systemic therapy linked with SBRT treatment?	If yes, answer each: Pre-SBRT, concurrent, planned post and indicate modality. See below for explanation of each.
TYPE OF SYSTEMIC THERAPY	Identify type of systemic therapy (may select more than 1 option)	If targeted therapy is selected, please identify type(s)

Baseline Pathologic Fracture

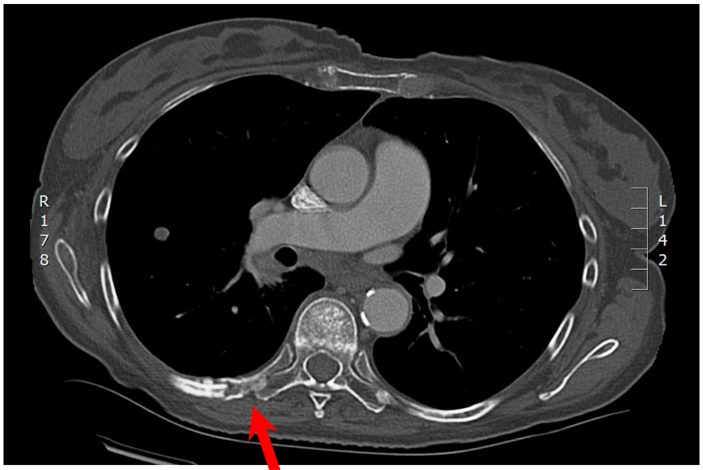

Soft Tissue Extension

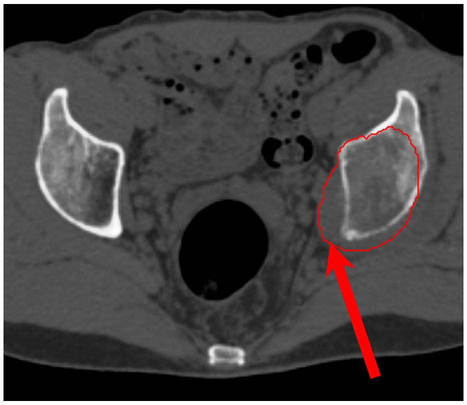

© 2020 Poon I. *JAMA Network Open*.

Systemic Therapy

- Pre-SBRT – Considered any systemic therapy that a patient is on immediately before SBRT. Example situations include when systemic therapy is given before SBRT in order to shrink the tumour to make it more amenable to treatment, or maintenance chemotherapy (eg. maintenance Pemetrexed in NSCLC) that is given before and after SBRT (should also be considered as Post-SBRT systemic) or targeted therapies that patients are on before and resume after SBRT. Select the time interval that corresponds to the break in systemic therapy prior to the start of SBRT: <1 week, 1-2 weeks, >2 weeks or unknown.
- Concurrent – the systemic agent is given during the course of SBRT – ie. there is no note of stopping the agent before the start of SBRT and remaining off for the duration of treatment or the SBRT is not intentionally scheduled between cycles of chemotherapy. If patients are on concurrent systemic therapy, please enter N/A in Pre-SBRT and Post-SBRT drop downs
- Post-SBRT – any systemic therapy that was planned to be given post SBRT –the decision to give chemotherapy is not based on response to treatment or a switch in agents based on response to treatment or appearance of new disease. Select the time interval that corresponds to the break in systemic therapy after completion of SBRT: <1 week, 1-2 weeks, >2 weeks or unknown.

SBRT TREATMENT DETAILS

Variable	Description	Comments
DOSE	Total dose prescribed (Gy)	Biological equivalent dose and single fraction equivalent dose will be calculated in spreadsheet using these values and assuming alpha/beta = 10
FX	Number of fractions	
START DATE	Date of Start of SBRT Treatment	
END DATE	Date of End of SBRT Treatment	Duration of the treatment will be calculated in the spreadsheet
GTV / ITV	Gross tumour volume (cc) measured on planning CT	Use the GTV for exhale phase if multiple GTVs are contoured.
CTV	Clinical target volume (cc) if applicable (from planning CT)	
PTV	Planning target volume (cc) (from planning CT)	
JOINT	Does the treatment volume include the joint of an extremity?	Answer for extremities only. Considered to include the joint if the PTV extends into joint space or is immediately adjacent to joint. See example below.
TECHNIQUE	Treatment delivery technique 3D, conformal arc, IMRT, VMAT, Cyberknife, other	

Joint

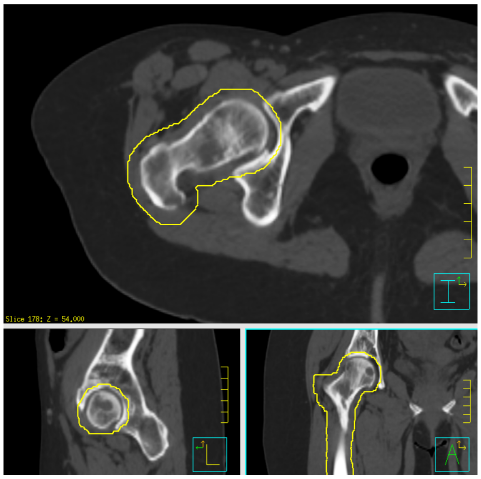

TARGET DOSIMETRIC DATA

Variable	Description	Comments
ITV/GTV MAX	Maximum dose to ITV (Gy) (GTV if ITV not available)	Report dose to GTV only if there is no ITV used.
ITV/GTV MIN	Minimum dose to ITV (GTV if ITV not available)	
ITV/GTV MEAN	Mean dose to ITV (GTV if ITV not available)	
PTV MAX	Maximum dose to PTV	
PTV MIN	Minimum dose to PTV	
PTV MEAN	Mean dose to PTV	

- If possible, tabular DVH data for PTV should be uploaded and relabeled (see instructions at the end of the guide)

LOCAL CONTROL – AFTER SBRT (additional field in Non-Spine Bone Mets Form)

Variable	Description	Comments
REGIONAL FAILURE	Did the patient fail in the same bone with a new metastasis appearing in a separate location? (Y/N?)	If yes, record date and details of regional failure

© 2020 Poon I. *JAMA Network Open*.

<u>Lymph Node-Soft Tissue Form Instructions</u>

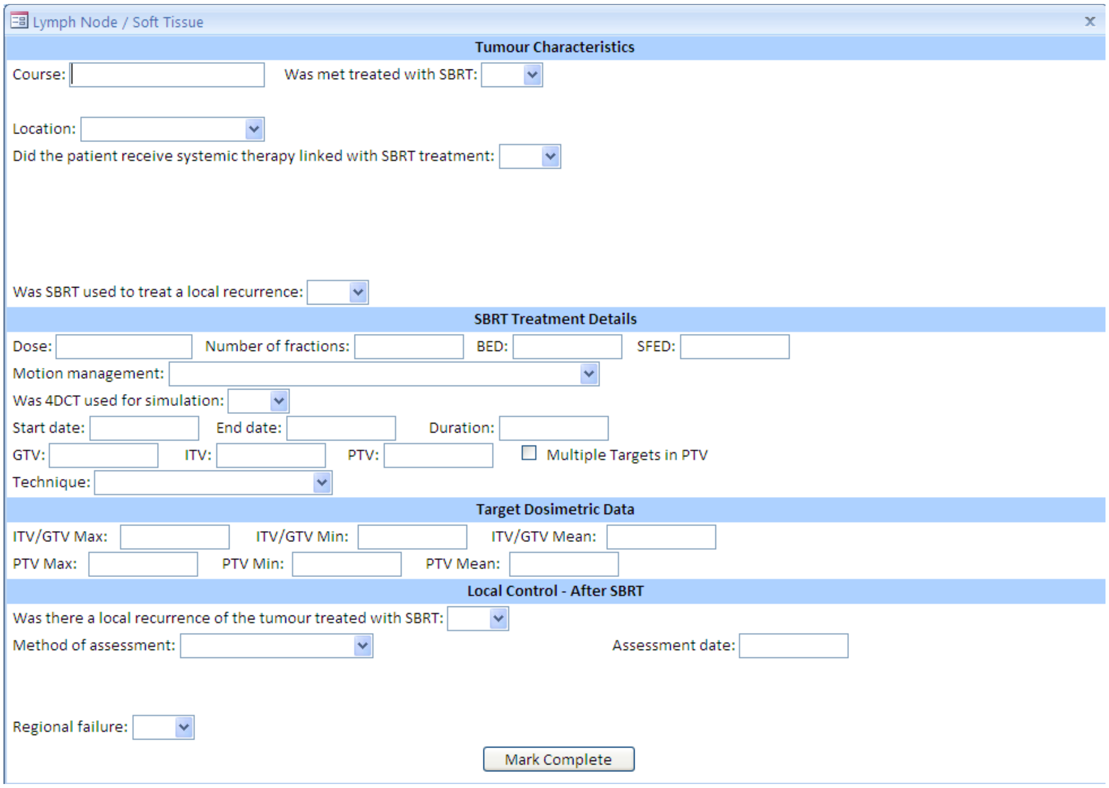

Lymph Node / Soft Tissue

Tumour Characteristics

Course: Was met treated with SBRT:

Location:

Did the patient receive systemic therapy linked with SBRT treatment:

Was SBRT used to treat a local recurrence:

SBRT Treatment Details

Dose: Number of fractions: BED: SFED:

Motion management:

Was 4DCT used for simulation:

Start date: End date: Duration:

GTV: ITV: PTV: Multiple Targets in PTV

Technique:

Target Dosimetric Data

ITV/GTV Max: ITV/GTV Min: ITV/GTV Mean:

PTV Max: PTV Min: PTV Mean:

Local Control - After SBRT

Was there a local recurrence of the tumour treated with SBRT:

Method of assessment: Assessment date:

Regional failure:

Mark Complete

TUMOUR CHARACTERISTICS

Variable	Description	Comments
COURSE	Number assigned to track each metastasis	eg. 2-4 first number represents the course number of SBRT and the second number represents the metastasis # (2nd course of SBRT but 4th metastasis)
SBRT	Was met treated with SBRT?(Y/N)	If no, indicate treatment modality and the course will be closed
LOCATION	H&N, Infraclavicular, Axillary,Hilar, Mediastinal, Mesenteric, Paraaortic, Common Iliaic, External Iliac, Internal Iliac, Inguinal, Other (specifiy) (drop down)	H&N includes cervical, supraclavicular, occipital and preauricular. Axillary includes pectoral, Inguinal includes femoral nodes. Please enter any additional site as other and specify location in free text box.
SYSTEMIC THERAPY	Did the patient receive systemic therapy linked with SBRT treatment?	If yes, answer each: Pre-SBRT, concurrent, planned post and indicatee modality. See below for explanation of each.
TYPE OF SYSTEMIC THERAPY	Identify type of systemic therapy (may select more than 1 option)	If targeted therapy is selected, please identify type(s)

© 2020 Poon I. *JAMA Network Open*.

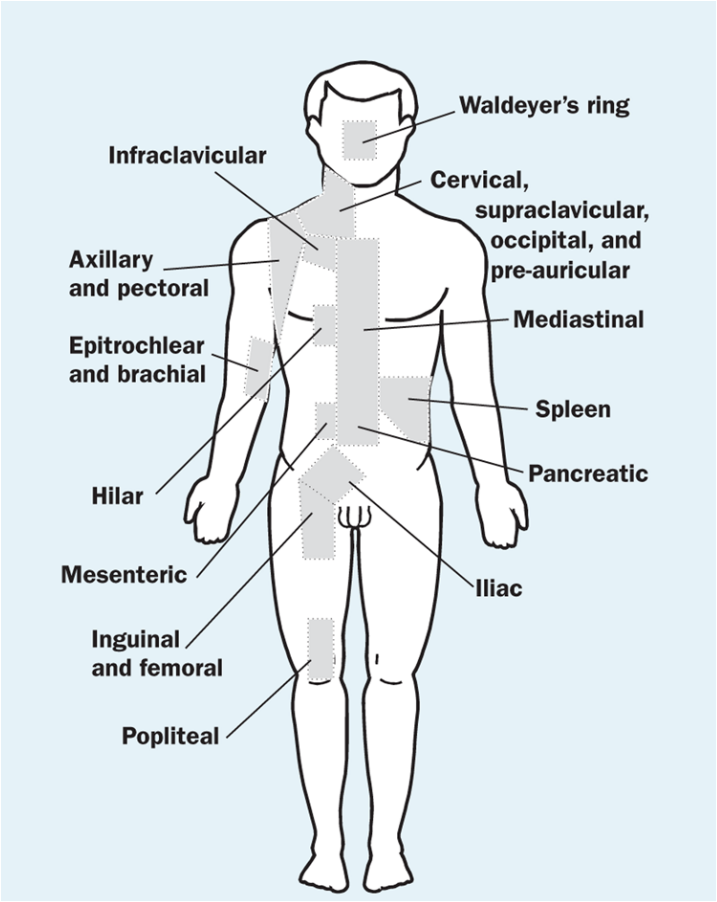

<u>Systemic Therapy</u>

- Pre-SBRT – Considered any systemic therapy that a patient is on immediately before SBRT. Example situations include when systemic therapy is given before SBRT in order to shrink the tumour to make it more amenable to treatment, or maintenance chemotherapy (eg. maintenance Pemetrexed in NSCLC) that is given before and after SBRT (should also be considered as Post-SBRT systemic) or targeted therapies that patients are on before and resume after SBRT. Select the time interval that corresponds to the break in systemic therapy prior to the start of SBRT: <1 week, 1-2 weeks, >2 weeks or unknown.
- Concurrent – the systemic agent is given during the course of SBRT – ie. there is no note of stopping the agent before the start of SBRT and remaining off for the duration of treatment or the SBRT is not intentionally scheduled between cycles of chemotherapy. If patients are on concurrent systemic therapy, please enter N/A in Pre-SBRT and Post-SBRT drop downs
- Post-SBRT – any systemic therapy that was planned to be given post SBRT –the decision to give chemotherapy is not based on response to treatment or a switch in agents based on response to treatment or appearance of new disease. Select the time interval that corresponds to the break in systemic therapy after completion of SBRT: <1 week, 1-2 weeks, >2 weeks or unknown.

SBRT TREATMENT DETAILS

Variable	Description	Comments
DOSE	Total dose prescribed (Gy)	Biological equivalent dose and single fraction equivalent dose will be calculated in spreadsheet using these values and assuming alpha/beta = 10
FX	Number of fractions	
MOTION MANAGEMENT	Indicate method of motion management: ABC, Abdominal Compression, Free Breathing/Nothing, Bodyfix, Gated, Other (specify) (drop down)	
4DCT	Was 4DCT used for simulation? (Y/N)	
START DATE	Date of Start of SBRT Treatment	
END DATE	Date of End of SBRT Treatment	Duration of the treatment will be calculated in the spreadsheet
GTV	Gross tumour volume (cc) measured on planning CT	Use the GTV for exhale phase if multiple GTVs are contoured.
ITV	Internal target volume (cc) if applicable (from planning CT)	If 2 lesions separate lesions make up the ITV, use half the volume for each entry.
PTV	Planning target volume (cc) (from planning CT)	
MULTIPLE TARGETS IN PTV	Y/N – more than one tumour is contoured as ITV/PTV	If 2 lesions separate lesions make up the PTV, use half the volume for each entry.
TECHNIQUE	Treatment delivery technique 3D, conformal arc, IMRT, VMAT, Cyberknife, other (specify) (drop down)	

TARGET DOSIMETRIC DATA

Variable	Description	Comments
ITV/GTV MAX	Maximum dose to ITV (Gy) (GTV if ITV not available)	Report dose to GTV only if there is no ITV used.
ITV/GTV MIN	Minimum dose to ITV (GTV if ITV not available)	
ITV/GTV MEAN	Mean dose to ITV (GTV if ITV not available)	
PTV MAX	Maximum dose to PTV	
PTV MIN	Minimum dose to PTV	
PTV MEAN	Mean dose to PTV	

- If possible, tabular DVH data for PTV should be uploaded and relabeled (see instructions at the end of the guide)

LOCAL CONTROL – AFTER SBRT (additional field in Lymph Node / Soft Tissue Form)

Variable	Description	Comments
REGIONAL FAILURE	Did the patient fail in the same or adjacent nodal chain with a new metastasis appearing in a separate location? (Y/N?)	If yes, record date and details of regional failure

© 2020 Poon I. *JAMA Network Open*.

Toxicities

- Toxicities are recorded as a separate tab
- All toxicities must be reviewed by the lead investigator at site
- When selecting site, please select the site of SBRT treatment that is most likely the cause of the side effect

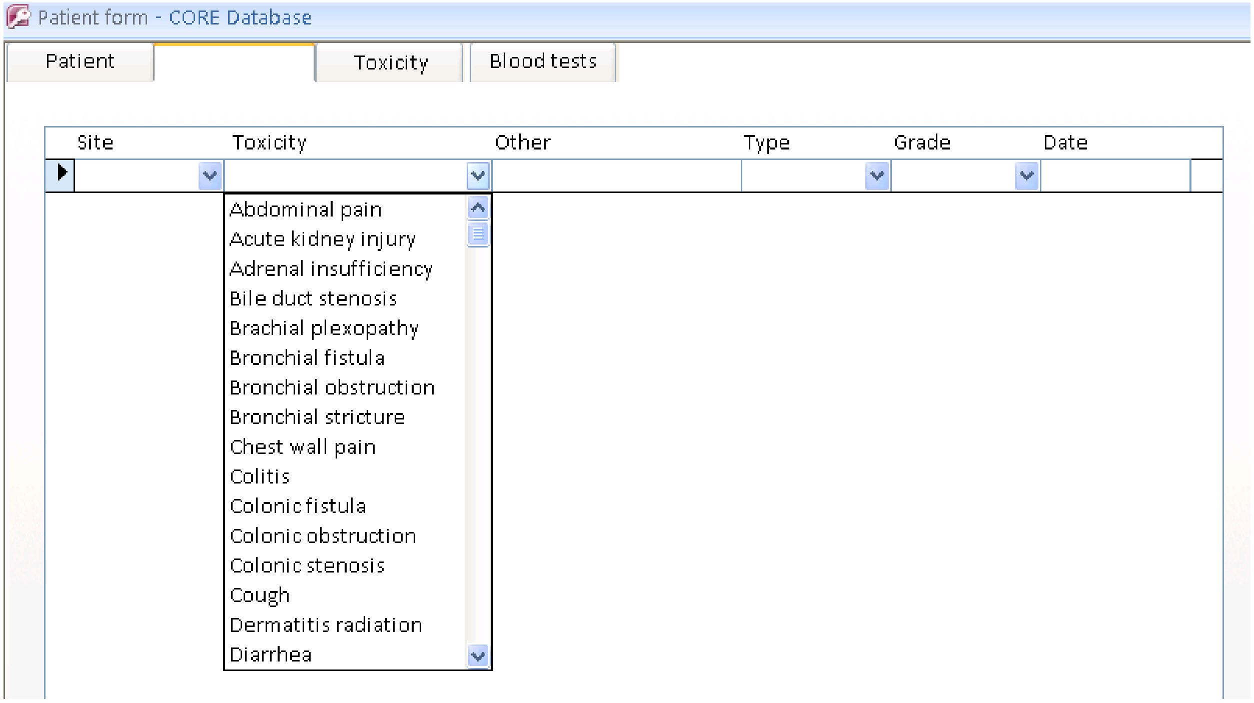

- Acute toxicities are those reported within 3 months of completion of SBRT
- Late toxicities are those reported >3 months after completion of SBRT
- Description of toxicities and grading as per CTAC v4
- Please record only Grade 3-5 toxicities

© 2020 Poon I. *JAMA Network Open*.

eFigure. Schematic outlining data quality assurance process and timeline.

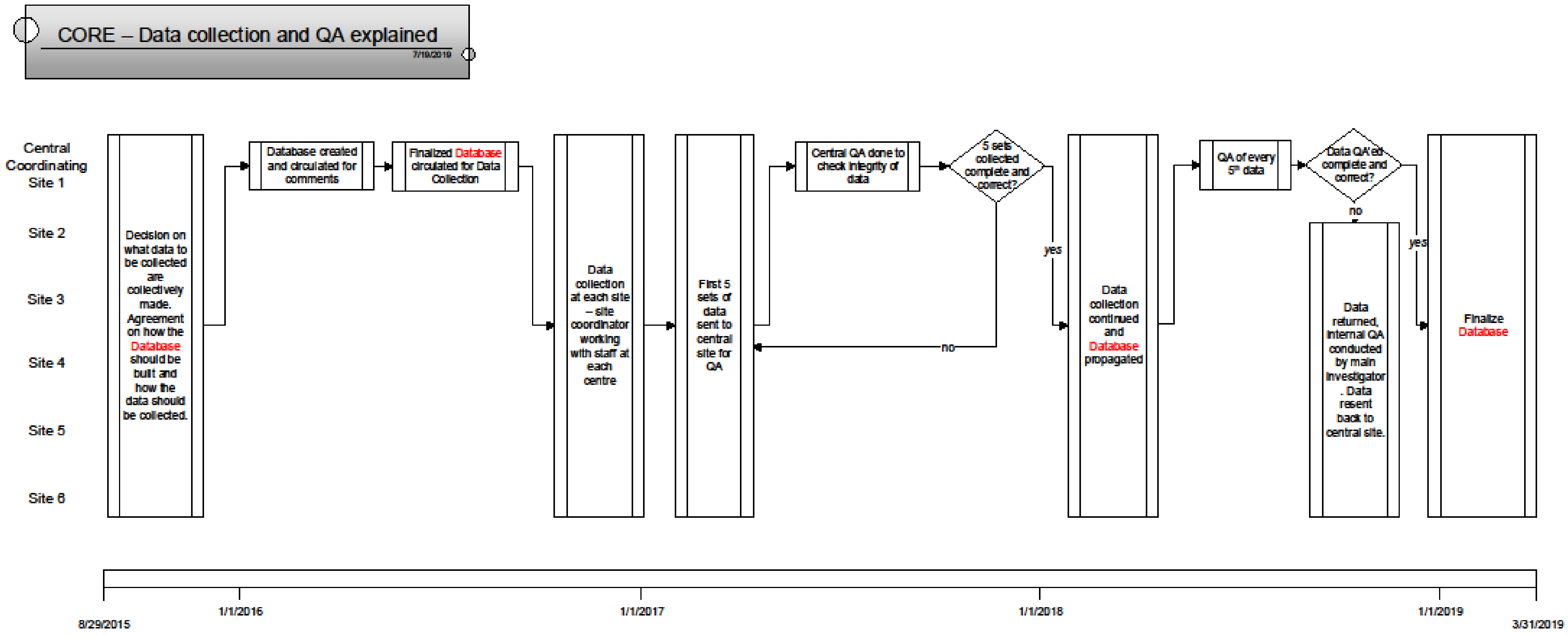

eTable 1. Characteristics of oligometastases treated with SBRT

	Total Number Lesions	Primary Cancer (frequency)	Crude Rate of Local Control (%)	Mean GTV/CTV (cm3) (range)	Mean ITV (cm3) (range)	Mean BED (Gy) (range)	# Fractions (frequency)	Mean Dose (Gy) (range)
Lung	648	Prostate (7)<br>Breast (19)<br>Colorectal (204)<br>Lung (202)<br>Kidney (34)<br>Others (182)	79.5	8.0<br>(0.2-147.5)	10.5<br>(0.4-169.6)	105.5<br>(37.5-151.2)	1 (92)<br>3 (33)<br>4 (242)<br>5 (233)<br>8 (16)<br>10 (30)<br>15 (2)	26.6 (26-30)<br>45.6 (24-54)<br>51.2 (40-60)<br>49.9 (25-60)<br>56.8 (48-60)<br>50(50-50)<br>52.5 (45-60)
Liver	185	Prostate (0)<br>Breast (13)<br>Colorectal (93)<br>Lung (14)<br>Kidney (2)<br>Others (63)	63.8	31.9<br>(0.1-261.8)	36.8<br>(0.4-343.7)	90.6<br>(28.8-180)	3 (30)<br>4 (1)<br>5 (114)<br>6 (33)<br>9 (1)<br>10 (6)	41.4 (18-60)<br>40 (40-40)<br>45.4 (28-60)<br>49.8 (30-60)<br>45 (45-45)<br>50 (50-50)
Spine	225	Prostate (58)<br>Breast (51)<br>Colorectal (4)<br>Lung (35)<br>Kidney (21)<br>Others (56)	74.1	43.3<br>(1.03-401.5)	n/a	58.1<br>(26.4-100)	1 (38)<br>2 (88)<br>3 (28)<br>4 (6)<br>5 (48)<br>7 (2)<br>10 (15)	19.7 (12-24)<br>25.0 (24-28)<br>25.5 (21-30)<br>30 (30-30)<br>35.1 (20-50)<br>36.5 (35-38)<br>49.3 (40-50)
Non-Spine Bone	194	Prostate (111)<br>Breast (19)<br>Colorectal (2)<br>Lung (24)<br>Kidney (13)<br>Others (25)	88.1	23.8<br>(0.2 – 382.6)	n/a	66.8<br>(37.5-106.4)	1 (14)<br>2 (8)<br>3 (25)<br>4 (2)<br>5 (105)<br>10 (40)	19.6 (15-28)<br>25.9 (24-31)<br>27.5 (24-30)<br>27.0 (24-30)<br>38.5 (25-50)<br>49.5 (30-50)
Adrenal	48	Prostate (1)<br>Breast (0)<br>Colorectal (2)<br>Lung (38)<br>Kidney (3)<br>Others (4)	72.9	27.1<br>(2.4-17.7)	29.4<br>(2.5-149.3)	72.1<br>(30-124.8)	3 (2)<br>4 (1)<br>5 (35)<br>6 (1)<br>9 (1)<br>10 (8)	52.0 (36-48)<br>20.0 (20-20)<br>39.5 (25-50)<br>30.0 (30-30)<br>45.0 (45-45)<br>50.0 (50-50)
Node/ Soft Tissue	116	Prostate (35)<br>Breast (3)<br>Colorectal (25)<br>Lung (13)<br>Kidney (6)<br>Others (34)	83.6	14.63<br>(0.3-113.0)	n/a	70.2<br>(35.7-115.5)	2 (1)<br>3 (17)<br>4 (3)<br>5 (58)<br>6 (3)<br>8 (1)<br>10 (32)<br>15 (1)	24.0 (24-24)<br>28.8 (21-36)<br>45.3 (40-48)<br>39.1 (25-55)<br>42.0 (30-48)<br>40.0 (40-40)<br>49.7 (40-50)<br>38.0 (38-38)

© 2020 Poon I. *JAMA Network Open*.

eTable 2. Combinations of Anatomic Sites treated with SBRT. Solitary lesions are indicated in brackets for each anatomic site. Three Site Combinations not included in diagram: Liver, Bone, Lymph/Soft Tissue (2), Lung, Adrenal, Lymph/Soft Tissue (2), Lung, Bone, Adrenal (1), Lung, Liver, Bone (2), Lung, Liver, Lymph/Soft Tissue (2)

	Lung	Liver	Bone	Adrenal	Soft Tissue
Lung	414 (252)	38	9	6	10
Liver		124 (76)	6	2	6
Bone			277 (195)	2	13
Adrenal				29 (26)	3
Soft Tissue					85 (47)

© 2020 Poon I. *JAMA Network Open*.

eTable 3. Survival statistics

	Stratification	Median (months) (95%CI)	1 yr (95%CI)	2 yr (95%CI)	3 yr (95%CI)	5 yr (95%CI)
Overall Survival		44.2 (39.2-48.8)	0.84 (0.82-0.86)	0.7 (0.66-0.72)	0.57 (0.53-0.60)	0.35 (0.30-0.40)
	Synchronous	33.2 (30.5-42.4)	0.79 (0.74-0.83)	0.61 (0.54-0.67)	0.48 (0.41-0.55)	0.33 (0.25-0.42)
	Metachronous ≤24 months	32.9 (28.8-39.9)	0.79 (0.74-0.83)	0.6 (0.55-0.66)	0.47 (0.41-0.54)	0.26 (0.19-0.34)
	Metachronous >24 months	53.6 (48.8-69.3)	0.91 (0.88-0.94)	0.83 (0.78-0.86)	0.7 (0.64-0.75)	0.44 (0.34-0.53)
	Breast	51.2 (> 42.4)	0.94 (0.86-0.97)	0.9 (0.81-0.95)	0.72 (0.59-0.82)	0.4 (0.22-0.57)
	Colorectal	49 (39.9-56.7)	0.89 (0.84-0.92)	0.76 (0.70-0.81)	0.67 (0.59-0.73)	0.36 (0.25-0.47)
	Kidney	81.9 (> 32.7)	0.83 (0.71-0.91)	0.73 (0.59-0.83)	0.64 (0.49-0.76)	0.57 (0.37-0.73)
	Lung	32.4 (28.8-38.5)	0.8 (0.74-0.84)	0.62 (0.55-0.68)	0.44 (0.37-0.51)	0.22 (0.15-0.3)
	Prostate	not reached	1 (1.0-1.0)	0.96 (0.90-0.98)	0.88 (0.76-0.94)	0.81 (0.60-0.92)
	Other Primary	26.3 (20.3-32.2)	0.73 (0.68-0.78)	0.51 (0.44-0.57)	0.39 (0.32-0.47)	0.3 (0.22-0.39)
	Lung Metastases Only	44.8 (38.5-49.7)	0.88 (0.84-0.91)	0.74 (0.69-0.78)	0.58 (0.52-0.63)	0.35 (0.28-0.42)
	Other Metastases	43.5 (37.5-51.2)	0.82 (0.78-0.84)	0.66 (0.62-0.70)	0.56 (0.51-0.61)	0.36 (0.29-0.43)
	Nodal/ Soft Tissue Metastases Only	not reached	0.96 (0.84-0.99)	0.76 (0.59-0.86)	0.76 (0.59-0.86)	0.65 (0.38-0.83)
	Other Metastases	43.5 (38.4-48.4)	0.84 (0.81-0.86)	0.69 (0.66-0.72)	0.56 (0.52-0.6)	0.35 (0.29-0.4)
Progression Free Survival		12.9 (11.6-14.2)	0.52 (0.49-0.55)	0.31 (0.28-0.34)	0.23 (0.20-0.26)	0.15 (0.12-0.18)
Widespread Progression		42.5 (36.8-53.5)	0.25 (0.22-0.27)	0.37 (0.34-0.40)	0.45 (0.41-0.49)	0.55 (0.50-0.59)

© 2020 Poon I. *JAMA Network Open*.

eTable 4. Description of Adverse Events.

Acute (≤3 months post SBRT)	Grade	Frequency	Treatment Site (N)
Bile Duct Stenosis	3	1	Lymph Node/Soft Tissue (1)
Cough	3	3	Lung (3)
Dermatitis	3	1	Spine (1)
Dysphagia	3	1	Spine (1)
Dyspnea	3	2	Lung (2)
Esophagitis	3	2	Lung (2)
Fatigue	3	5	Spine (2), Lung (2), Bone (1)
Fracture	3	2	Spine (2)
Hemorrhage	3	1	Spine (1)
Pain	3	6	Liver (1), Adrenal (1), Lung (1), Spine (3)
Pneumonitis	3	5	Lung (5)
	4	1	Lung (1)
	5	1	Lung (1)
**Late (>3 months post SBRT)**			
Bile Duct Stenosis	4	1	Liver(1)
	5	1	Liver (1)
Brachial Plexopathy	3	1	Spine (1)
Bronchial Stricture	3	1	Lung (1)
Cough	3	5	Lung (5)
Dyspnea	3	3	Lung (3)
Esophagitis	3	1	Lung (1)
Fracture	3	4	Spine (1), Bone (3)
Liver Abscess	3	2	Liver (2)
Pain	3	7	Lung (6), Bone (1)
Pneumonitis	3	8	Lung (8)
Radiculitis	3	1	Spine (1)

© 2020 Poon I. *JAMA Network Open*.
